# Chinese Herbal Medicines for Diabetic Cardio‐Cerebrovascular Diseases: Key Bioactive Metabolites and Action Mechanisms

**DOI:** 10.1155/jnme/5563248

**Published:** 2026-02-24

**Authors:** Ying Su, Shuwen Luo, Wei Li, Cuicui Cheng, Wei Liu, Xinyu Yang, Zhen Xing

**Affiliations:** ^1^ Department of Traditional Chinese Medicine, Dongping People’s Hospital, Taian, 271500, Shandong, China; ^2^ Department of Traditional Chinese Medicine, Hainan Province Anning Hospital, Haikou, 572299, Hainan, China; ^3^ Linhe Kangyou Hospital, Bayannur, 015099, Inner Mongolia, China; ^4^ Institute of Basic Research in Clinical Medicine, China Academy of Chinese Medical, Beijing, 100700, China; ^5^ Dongfang Hospital, Beijing University of Chinese Medicine, Beijing, 100078, China, bucm.edu.cn

**Keywords:** apoptosis, Chinese herbal medicine, diabetic cardio-cerebrovascular disease, inflammation, mitochondria, oxidative stress

## Abstract

Currently, the global incidence of diabetes is increasing, particularly in populous developing regions. In China, over 290 million people are affected by diabetic cardio‐cerebrovascular diseases. These diseases account for more than 40% of deaths and impose a significant economic burden on both society and families. Diabetes can result in vascular complications through multiple mechanisms, including cardiovascular and cerebrovascular diseases. Current management guidelines recommend conducting risk assessments before prescribing medications like antihypertensives, hypoglycemics, and lipid‐lowering drugs, alongside lifestyle interventions, to help prevent cardio‐cerebrovascular diseases. However, pharmacological approaches have several limitations, including adverse drug reactions and variability in patient responses. Chinese herbal medicine (CHM) exerts its therapeutic effects via bioactive metabolites that modulate multiple molecular targets, including enzymes, receptors, and transcriptional regulators, through complex interactions with cellular signaling networks. While modern pharmacological research validates its polypharmacological mechanisms, concerns persist regarding potential botanical drug interactions, toxicological profiles, and pharmacokinetic variability of certain botanicals. Only through a balanced scientific approach can CHM’s unique therapeutic value be fully realized. This review evaluates the efficacy of CHM in mitigating metabolic disorders, focusing on its diverse pharmacological mechanisms, including antioxidant defenses, inflammation suppression, and programmed cell death regulation. It elucidates the role of pivotal signaling cascades, including the glucagon (GLC)/5′‐adenosine monophosphate–activated protein kinase (AMPK)/nuclear transcription factor‐κB (NF‐κB) axis, the GLC/peroxisome proliferator–activated receptor α (PPARα)/PGC‐1α pathway, as well as the PI3K/Akt and AMPK/mammalian target of rapamycin (mTOR) signaling pathway, alongside oxidative stress and inflammatory responses. However, future research should prioritize well‐structured clinical trials and mechanistic studies to substantiate CHM’s therapeutic potential.

## 1. Introduction

In recent decades, the global diabetes incidence has soared, rising steadily with population growth [1]. By mid‐2035, the global diabetic population will surpass 592 million [2]. Diabetes can lead to vascular complications through both direct and indirect mechanisms. Macrovascular lesions may contribute to cardiovascular, cerebrovascular, and peripheral vascular diseases. According to WHO statistics, 32% of all global deaths (equivalent to 17.9 million lives annually) are attributable to cardio‐cerebrovascular diseases, making them the foremost mortality factor. In China, diabetic cardio‐cerebrovascular diseases have been diagnosed in more than 290 million patients, representing over 40% of mortality cases and placing heavy economic pressure on both familial and societal levels [3]. This underscores the urgent need to discover safe and effective new therapies to alleviate the burden of diabetic cardio‐cerebrovascular diseases on global health and longevity.

Current management guidelines recommend conducting risk assessments before prescribing medications such as antihypertensives, hypoglycemics, and lipid‐lowering agents, alongside implementing lifestyle interventions to help prevent new cases of cardio‐cerebrovascular diseases [4–6]. However, pharmacological approaches have several limitations, including adverse drug reactions and variability in patient responses [7, 8]. Research indicates that metabolic disturbances, inflammation, and oxidative stress are key initiators or mediators of cardiac and vascular injury in the development of cardio‐cerebrovascular diseases [9–11]. Oxidative stress, inflammation, and apoptosis interact in a self‐sustaining vicious cycle [12]. The cycle begins with excessive reactive oxygen species (ROS) production, which not only activates inflammatory pathways (e.g., NF‐κB) but also induces mitochondrial dysfunction. This oxidative damage subsequently triggers inflammatory responses and excessive cell death. Inflammation further aggravates oxidative stress through mechanisms such as respiratory bursts in immune cells and the release of proinflammatory cytokines, which also directly stimulate apoptosis. Meanwhile, heightened apoptotic activity releases pro‐oxidant molecules and damage‐associated molecular patterns, reinforcing oxidative stress and inflammation. Together, these interconnected processes contribute to vascular endothelial injury, progressive loss of cardiomyocytes/neurons, and pathological tissue remodeling, ultimately accelerating disease progression [13].

Therefore, identifying novel targets that play pivotal roles in the pathogenesis of cardio‐cerebrovascular diseases could facilitate the discovery and development of more effective and safer treatment options. The clinical applications of Chinese herbal medicine (CHM) are substantiated by the pharmacological activities of its bioactive metabolites, which exert therapeutic effects through multitarget interactions with cellular signaling pathways and biological networks [14]. Modern research has demonstrated that these metabolites modulate key molecular targets, including enzymes, receptors, and gene expression regulators, resulting in systemic therapeutic outcomes. For example, flavonoids (e.g., puerarin [Pue], baicalin) from kudzu root and ginkgo leaves dilate blood vessels, regulate lipids, and combat ischemia–reperfusion injury; saponins (e.g., ginsenosides, astragaloside IV) in ginseng and astragalus protect myocardium, inhibit platelet aggregation, and prevent remodeling [15]. In this study, we summarized the effectiveness of CHM in the treatment of diabetic cardio‐cerebrovascular diseases, and the roles of CHM active metabolites and their potential mechanisms. While pharmacodynamic synergies and network pharmacology provide a scientific framework for CHM’s efficacy, standardization and mechanistic elucidation remain critical challenges in its integration with contemporary medical paradigms. Thus, future research should prioritize well‐structured clinical trials and mechanistic studies to substantiate CHM’s therapeutic potential.

### 1.1. Pathogenesis of Cardio‐Cerebrovascular Diseases in Diabetes Mellitus (DM)

The seminal hypothesis proposed by Ross and Glomset regarding the pathogenesis of atherosclerosis posits that this vascular pathology initiates as a reactive process to endothelial “injury” in arterial walls [16]. This pathological cascade involves a series of events, including endothelial desquamation, platelet adhesion, and aggregation, followed by platelet degranulation at sites of endothelial denudation. These responses to endothelial damage constitute the fundamental mechanisms underlying atherogenesis [17]. Hyperglycemia‐mediated endothelial dysfunction is primarily driven by the downregulation of circular homeodomain‐interacting protein kinase 3 RNA, a critical regulatory mechanism in diabetic vascular complications. In addition, oxidized low‐density lipoprotein (ox‐LDL) impairs endothelial cell function through multiple pathways, including inhibition of cholesterol efflux, activation of the apoptosis signal–regulated kinase 1/nucleotide‐binding oligomerization domain (NOD)–like receptor (NLR) family pyrin domain–containing protein 3 (NLRP3) inflammasome signaling cascade, and induction of endoplasmic reticulum stress [9]. Collectively, these molecular mechanisms contribute to endothelial injury, the central pathophysiological process driving the development of cardio‐cerebrovascular diseases in patients with DM.

Patients with DM often experience pathological conditions, such as prolonged hyperglycemia and metabolic disturbances, which can result in long‐term damage to the blood vessel endothelium [18]. Hyperglycemia and metabolic disturbances increase oxidative stress, further exacerbating endothelial injury. The inflammatory cascade involves increased production of key mediators including proinflammatory cytokines (notably TNF‐α, IL‐6, and MCP‐1) along with elevated expression of cellular adhesion molecules (ICAM‐1, VCAM‐1, and selectins). These mediators enhance inflammatory cell adhesion to the endothelium, followed by transmigration into the vessel wall. Once within the vascular wall, mononuclear leukocytes adhere to the vascular surface, traverse the endothelial layer, and differentiate into macrophages. These macrophages then phagocytize LDL cholesterol (LDL‐C), which is elevated in diabetic patients, particularly those with hyperlipidemia. Subsequently, these cells transform into foam cells.

During the inflammatory response, blood platelets aggregate and adhere to the vascular wall, while smooth muscle cells proliferate and migrate into the endothelium. As foam cells degenerate and undergo necrosis, the lipids within them are released into the vascular wall, leading to the formation of an extracellular lipid core. When the lipid core becomes significantly enlarged and macrophages predominate the area, an inflammatory response is triggered. The inflammatory cascade activates NF‐κB signaling, releasing proinflammatory cytokines (TNF‐α, IL‐6) that simultaneously drive insulin resistance, β‐cell dysfunction, and endothelial damage. Parallel apoptosis triggered by mitochondrial and death receptor pathway dysregulation exacerbates metabolic and vascular injury through β‐cell and endothelial cell loss. These interconnected processes form a vicious cycle, critically advancing DM‐related cardio‐cerebrovascular pathogenesis. Additionally, oxidative stress, characterized by an imbalance in redox status, is marked by excessive production of ROS and impaired antioxidant defense systems [19]. In DM, excessive ROS impair vascular endothelial integrity, leading to junctional dysfunction, enhanced vascular permeability, and consequent disease exacerbation [20].

### 1.2. Therapeutic Mechanism of CHM in Treating Diabetic Cardiovascular Diseases

#### 1.2.1. Metabolic Pathway

##### 1.2.1.1. Herbal Formula

**FIGURE 1:**
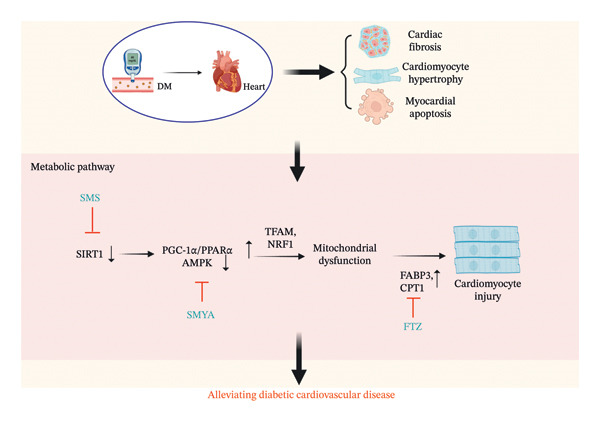
The mechanism of Chinese herbal medicine in treating diabetic cardiovascular diseases through the metabolic pathway. DM, diabetic mellitus; SMS, ShengMai‐San; SIRT1, sirtuin 1; AMPK, 5′‐adenosine monophosphate–activated protein kinase; PGC‐1α, proliferator‐activated receptor gamma coactivator 1‐alpha; SMYA, Si‐Miao‐Yong‐An decoction; PPARα, peroxisome proliferator–activated receptor α; FABP3, fatty acid binding protein 3; CPT1, carnitine palmitoyl transferase 1; FTZ, Fufang‐Zhenzhu‐Tiaozhi capsule.

ShengMai‐San (SMS) is a traditional herbal formulation composed of *Panax quinquefolius L.*, *Ophiopogon japonicus* (Thunb.) Ker Gawl., and *Schisandra chinensis* (Turcz.) Baill. and has been widely employed in the treatment of ischemic diseases. Recent research investigated the cardioprotective properties of SMS in diabetic cardiomyopathy (DCM), particularly through the enhancement of mitochondrial lipid metabolism [21]. The experimental design employed db/db mice (leptin receptor‐deficient) as the diabetic model, with matched C57BLKS mice serving as controls. Cell vitality, mitochondrial membrane potential (ΔΨm), ATP levels, and activity of the oxidative phosphorylation complex were measured in the palmitic acid‐activated H9C2 cells. Additionally, the sirtuin 1 (SIRT1)/AMPK/peroxisome proliferator–activated receptor gamma coactivator 1‐alpha (PGC‐1α) pathway and mitochondrial uncoupling pathway were analyzed by Western blotting and real‐time quantitative reverse transcription PCR (RT‐qPCR) [22]. The db/db mice displayed severe hyperglycemia, marked obesity, profound hyperlipidemia, and significant impairment in myocardial function. SMS administration effectively reversed diabetes‐induced myocardial dysfunction [23] and restored mitochondrial structure and function. Moreover, SMS markedly upregulated the SIRT1 and phosphorylated AMPKα protein expression, reducing the levels of uncoupling protein 2 and acetylated PGC‐1α. SMS administration also restored the expression of TFAM and NRF1 in both H9C2 cells and diabetic myocardial tissue. Taken together, these results demonstrate that SMS ameliorates diabetes‐induced cardiac dysfunction by enhancing mitochondrial lipid metabolic capacity (Figure 1 and Table 1).

**TABLE 1:** Therapeutic mechanism of Chinese herbal medicine in treating diabetic cardio‐cerebrovascular disease.

Event	Classification	Chinese herbology	Type of CHM	Metabolites	Dose	Duration	Control	Type of study	Model	Indicators	Effects	Refs
Cardiovascular disease	Metabolic pathway	SMS	Herbal formula	*Panax quinquefolius* L., *Ophiopogon japonicus* (Thunb.) Ker Gawl., Schisandra chinensis (Turcz.) Baill.	3 g/kg, 4.5 g/kg	2 months	Negative	In vivo	Mice	SIRT1↑, AMPK↑, PGC‐1α↓	Mitochondrial lipid metabolism	[21]
		SMYA	Herbal formula	LJF, SR, ASR, and GReR	12.29 g/kg/d	15 weeks	Positive met	In vivo	Mice	PGC‐1α↓, PPARα↓, AMPK↑	Glucolipid metabolism	[24]
		FTZ	Herbal formula	Atractylodis macrocephalae rhizoma, Salviae miltiorrhizae radix et rhizoma, Notoginseng radix et rhizoma, Coptidis rhizome, and Cirsii japonici herba et radix	0.6 g/kg/d, 1.2 g/kg/d and 2.4 g/kg/d	12 weeks	Positive met	In vivo	Mice	FABP3↓, CPT1↓	Lipid metabolism	[25]
	Intracellular inflammatory pathway	*Salvia miltiorrhiza* Bunge	Monomer	SAA CF: C_26_H_22_O_10_	0.5 mg/kg, 1 mg/kg	6 weeks	Positive atorvastatin ATV	In vivo	Rats	hs‐CRP↓, NLRP3↓, NF‐κB↓	Anti‐inflammatory effects	[26]
		*Salvia miltiorrhiza* Bunge	Monomer	TSN CF: C_19_H_18_O_3_	5 mg/kg	NA	Positive wortmannin	In vivo	Rats	NF‐*κ*B↓, TNF‐α↓, IL‐6↓	Alleviating inflammation	[27]
		CHE	Monomer	benzo [c] phenanthridine alkaloid CF: C_21_H_18_NO_4_	5 mg/kg	NA	Positive PAG	In vivo	Rats	CSE↑, PKC↓, NF‐κB↓	Alleviating inflammation	[28]
		*Pueraria* DC.	Monomer	Kakonein CF: C_21_H_20_O_9_	20, 40, or 80 mg/kg	7 days	Positive metformin	In vivo	Mice	NLRP3↓	Anti‐inflammatory effects	[29]
		*Phelipanche astragali* (Mouterde) Holub	Monomer	APS CF: C_10_H_7_ClN_2_O_2_S	1, 2 g/kg	10 weeks	Positive insulin	In vitro	Hamsters	MMP‐2↓	Anti‐inflammatory effects	[30]
		*Phelipanche astragali* (Mouterde) Holub	Monomer	Ferulic acid and astragaloside IV CF: CH_3_OC_6_(OH)CHCHCOOH; C_41_H_68_O_14_	50 mg/kg/d	10 weeks	Positive metformin	In vivo	Rats	MCP‐1↓, TNF‐α↓, NF‐κB↓	Inhibitory inflammatory pathway	[31]
		*Tripterygium* Hook.f.	Monomer	TwHF CF: C_20_H_24_O_6_	50, 100, or 200 μg/kg/d	8 weeks	Negative	In vivo	Rats	MCP‐1↓, TNF‐α↓, IL‐1β↓, NF‐κB↓	Anti‐inflammatory effects	[32, 33]
		Schisandra chinensis	Monomer	Sch B CF: C_23_H_28_O_6_	20, 40 mg/kg; 2.5, 5, 10 μm	8 weeks; 36, 72 h	Negative	In vivo In vitro	Mice Cells	MyD88↓	Anti‐inflammatory effects	[34]
		AED	Monomer	AED CF: C_15_H_10_O_5_	50, 100 mg/kg/d	4 weeks	Positive glyburide	In vivo	Rats	NLRP3↓	Anti‐inflammatory effects	[35]
		*Lycium chinense*	Monomer	LCME CF: C_11_H_14_N_2_O	100, 400 mg/kg	5 weeks	Negative	In vivo	Rats	IL‐1β↓, TNF‐α↓, IL‐6↓, NF‐κB↓	Inhibitory inflammatory pathway	[36]
		*Pueraria*	Monomer	Pue CF: C_21_H_20_O_9_	100, 150 mg/kg	21 days	Negative	In vivo In vitro	Rats, cells	NLRP3↓, IL‐1β↓, IL‐18↓	Anti‐inflammatory effects	[37]
		*Panax notoginseng*	Monomer	NR1 CF: C_47_H_80_O_18_	10, 20, 40, 80, 160 μM	72 h	Negative	In vitro	Cells	TNF‐α↓, IL‐6↓, IL‐10↓	Anti‐inflammatory effects	[38]
		*Myrica cerifera*	Monomer	Myr CF: C_21_H_20_O_12_	75, 150, 300 mg/kg/d; 6.25, 12.5, 25 μg/mL	8 weeks; 12, 24, and 36 h	Negative	In vivo In vitro	Mice Cells	NF‐κB↓	Inhibitory inflammatory pathway	[39]
		*Prunella* L	Single medicinal botanical drug	Prunella	NA	NA		NA	NA	IL‐6↓	Inhibitory inflammatory pathway	[40]
		*Anoectochilus roxburghii* (Wall.) Lindl.	Single Chinese botanical drug	L‐rhamnose, L‐arabinose, D‐xylose, D‐mannose, D‐glucose, and D‐galactose	100 or 300 mg/kg	15 days	Positive metformin	In vivo In vitro	Mice, Cells	MCP‐1↓, ICAM‐1↓, MMPs↓, NF‐κB↓, p38↓	Alleviating inflammation	[41]
		*Dendrobium officinale* Kimura & Migo	Single Chinese botanical drug	DOE	75, 150, 300 mg/kg	8 weeks	Negative	In vivo	mice	TNF‐α↓, IL‐1β↓, NF‐κB↓	Inhibitory inflammatory pathway	[42]
		FTZ	Herbal formula	Rhizoma coptidis, Radix Salvia Miltiorrhiza, Radix Notoginseng, Fructus Ligustri Lucidi, Herba Cirsii Jeponici, Cortex Eucommiae, Fructus Citri Sarcodactylis, and Radix Atractylodes Macrocephala	1.2 g/kg/d	16 weeks	Negative	In vivo	Pigs	PI3K↑, AKT↑, p‐AKT↑, p‐NF‐κB↓, NF‐κB↓, IL‐6↓, TNF‐α↓	Inhibitory inflammatory pathway	[43]
		JLD	Chinese patent medicine	Panax, Polygonatum, Atractylodes, Sophora, Ophiopogon, Rehmannia, Reynoutria, Cornus, Poria cocos, Eupatorium, Coptis, Anemarrhena, Epimedium, Salvia, Pueraria, Litchi, Lycium	3.5 g/kg/d	8 weeks	Positive dapagliflozin	In vivo In vitro	Mice, Cell	TP53↓, TNF↓, TGFβ1↓	Alleviating inflammation	[44]
	Oxidative stress pathway	*Andrographis paniculata* (Burm.f.) Wall. ex Nees	Monomer	Andro CF: C_20_H_30_O_5_	1, 10, or 20 mg/kg/d	12 weeks	Negative	In vivo In vitro	Mice Cells	ROS↓, NOX↓, Nrf2↑	Inhibitory OS pathway	[45]
		*Salvia miltiorrhiza* Bunge	Monomer	SAA CF: C_26_H_22_O_10_	0, 1, 5, 10, 25, 50, 100 μmol/L	1, 3, and 7 day(s)	Negative	In vitro	Cells	eNOS↑, AKT↑	Inhibitory OS pathway	[46]
		Erigeron breviscapus	Monomer	Breviscapine CF: C_21_H_18_O_12_	20 mg/kg	4 weeks	Negative	In vivo	Rats	MDA↓, SOD↑, GSH‐Px↑	Inhibitory OS pathway	[47]
		*Astragalus mongholicus* Bunge	Monomer	APS CF: C_10_H_7_ClN_2_O_2_S	0.1, 1, 10, 100 μg/mL	0, 12, 24, 48, 72 h	Negative	In vitro	Cells	MDA↓, SOD↑, GSH‐Px↑	Antioxidation effect	[48]
		ASI	Monomer	ASI CF: C_48_H_78_O_20_	10 mg/kg/d	3 weeks	Positive CC	In vivo	Mice	AMPK↑, Nrf2↑	Inhibitory OS pathway	[49]
		*Curcuma long*a L.	Monomer	L3 CF: C_19_H_16_O_4_	0.5, 1, 2 mmol/kg/d	16 weeks	Negative	In vivo	Mice	MDA↓, SOD↑, GSH↑	Antioxidation effect	[50]
		*Ophiopogon japonicus* (Thunb.) Ker Gawl.	Monomer	OJP1 CF: C_44_H_70_O_16_	100, 200, 300 mg/kg	28 days	Positive metformin	In vivo	Rats	MDA↓, CAT↑, SOD↑, GPx↑	Antioxidation effect	[51]
		*Panax quinquefolius* L.	Monomer	Ginsenoside Rb1 CF: C_59_H_100_O_27_	0, 3, 10, 30 μM; 50 mg/kg/d	4 weeks	Negative	In vivo In vitro	Mice Cells	NOX2↓, Nrf2↑	Antioxidation effect	[52]
		*Panax quinquefolius* L.	Monomer	PNS CF: C_47_H_80_O_17_	100, 200 mg/kg	12 weeks	Positive metformin	In vivo	Mice	Modulating ROS	Antioxidation effect	[53]
		*Aralia taibaiensis* Z. Z. Wang	Monomer	sAT CF: C_5_H_10_O_5_	25, 50, 75 μg/mL	24 h	Negative	In vitro	Cells	Modulating ROS, Nrf2↑	Antioxidation effect	[54]
		*Scutellaria baicalensis* Georgi, *Curcuma longa* L	Herbal formula	Cur and Bai	75, 150 mg/kg; 1.62, 3.25, 7.50, and 15 mg/mL	4 weeks; 24 h	Negative	In vivo In vitro	Rats Cells	Modulating ROS	Inhibitory OS pathway	[55]
		THJ	Herbal formula	Persicae semen, Polygonatum sibiricum, Carthami flos	0.125, 0.25, 0.5 g/kg/d	12 weeks	Negative	In vivo	mice	Modulating ROS, MDA↓, SOD↑, GSH‐Px↑	Inhibitory OS pathway	[56]
		YNJ	Herbal formula	Gypsum, Rehmanniae Radix Praeparata, Anemarrhenae Rhizoma, Ophiopogonis Radix, Achyranthis Bidentatae Radix	4.52 g/kg/d	10 weeks	Negative	In vivo	Rats	SIRT1↑, Nrf2↑, NQO1↑	Inhibitory OS pathway	[57]
		SMS	Herbal formula	*Panax ginseng*, *Ophiopogon japonicus*, *Schisandra chinensis*	1.8 g/kg/day	10 weeks	Negative	In vivo	Rats	NOX2↓, NOX4↓	Inhibitory OS pathway	[58, 59]
		Huayu Tongmai Granules	Herbal formula	NA	3 mg/kg	12 weeks	Negative	In vivo In vitro	Rats Cells	Modulating ROS, NO↑	Antioxidation effect	[60]
		WXKL	Chinese patent medicine	Codonopsis, Rhizoma Polygonati, Notoginseng, Amber, Nardostachys	3 g/kg; 1, 3 g/L	8 weeks; 24 h	Negative	In vivo In vitro	Rats Cells	Modulating ROS	Antioxidation effect	[61]
		SFI	Chinese patent medicine	NA	10 mL/kg	2 h	Positive Wortmannin	In vivo	Rats	MDA↓, SOD↑	Antioxidation effect	[62]
	Autophagy	Resveratrol	Monomer	Resveratrol CF: C_14_H_12_O_3_	60, 300 mg/kg/d	4 weeks	Positive bafilomycin A1	In vivo In vitro	Mice Cells	SIRT1↑, Rab7↑	Inhibitory autophagy pathway	[63]
		GSC	Herbal formula	Panax ginseng, Panax notoginseng, Ligusticum chuanxiong	50, 100, 200 mg/L	48 h	Positive metformin	In vitro	Cells	mitophagy↓	Antiautophagy effect	[64]
		SJTYD	Herbal formula	Radix Astragali seu Hedysari, Radix Platycodonis, Radix Bupleuri, Rhizoma Anemarrhenae, Fructus Corni, Rhizoma Sparganii, Rhizoma Cimicifugae, and Herba Leonuri	50 mg/kg/d	4 days	Negative	In vivo	Mice	mTOR↑, LC3A‐II↑	Antiautophagy effect	[65]
	Apoptosis	*Astragalus mongholicus* Bunge	Monomer	Astragaloside IV CF: C_41_H_68_O_14_	0.5, 5.0, 50 μg/mL	48 h	Positive RSG	In vitro	Cells	Apoptotic nuclei↑	Promotes Apoptosis	[66]
		*Salvia miltiorrhiza* Bunge	Monomer	SAA CF: C_26_H_22_O_10_	3 mg/kg	6 weeks	Positive metformin	In vivo	Rats	Bax↑, Bcl‐2↑, Caspase3↓, Caspase9↓	Apoptosis inhibition	[67]
		Huangqi‐Danshen metabolite, HDC	Herbal formula	Licorice, Hedysarum multijugum, Santalum album L., Aurantii Fructus Immaturus, Platycladi Semen, Radix Salviae, Trichosanthis Radix, hirudo, Folium Nelumbinis, Rehmanniae Radix Praeparata, Panax ginseng, Poria cocos.	6, 12, and 24 g/kg	10 weeks	Positive Gliclazide	In vivo	Rats	Bcl‐2↑	Antiapoptotic effect	[68]
		ZGJTSXF	Herbal formula	Panax ginseng, Astragalus membranaceus, Rehmannia glutinosa, Pueraria lobata, Cornus officinalis, Salvia miltiorrhiza, Coptis chinensis, *Ophiopogon japonicus*, Crataegus	16.84, 33.67, 67.34 g/kg/d	4 weeks	Positive metformin	NA	NA	Bcl‐2↑, Bcl‐xL↑	Apoptosis inhibition	[69]
		QGQXM	Herbal formula	Huangqi, Danggui, Danshen, Chuanxiong, Gegen, Shanzhuyu, Hongjingtian, Yinyanghuo	12 g/kg/d	4 weeks	Positive metformin	In vivo	Rats	Bcl‐2↑, Caspase9↓	Antiapoptotic effect	[70]
		FTZ	Herbal formula	*Coptidis Rhizoma, Ligustri Lucidi Fructus, Salviae miltiorrhizae radix et Rhizoma, Cirsii japonici herba, Eucommiae cortex, Citri sarcodactylis Fructus, Notoginseng Radix et Rhizoma, and Atractylodes macrocephala Rhizoma*	1.2 g/kg; 5, 20, 50 μg/mL	22 weeks; 24 or 48 h	Positive Metformin Atorvastatin	In vivo In vitro	Minipig Cells	Bax↑, Caspase 3↓, Bcl‐2↑	Alleviating apoptosis	[71]
		*Cornus officinalis* Siebold & Zucc., *Dioscorea oppositifolia* L.	Herbal formula	Mor, Dio	25, 50, or 100 μg/mL	24 or 72 h	Positive Metformin	In vitro	Cells	Bax↑, Bcl‐2↑	Alleviating apoptosis	[72]
		QSYQ	Chinese patent medicine	Danshen, Sanqi, Huangqi, Jiangxiang	NA	24 h	Positive LY294002	In vitro	Cells	caspase‐3↓, Bax↑, Bcl‐2↑	Antiapoptotic effect	[73]
		HJT	Chinese patent medicine	Rhodiola Wallichiana	10, 20, 40, 80, 160, or 320 mL/L	48 h	Negative	In vitro	Cells	Bax↑, Bcl‐2↑	Alleviating apoptosis	[74]
	Others	*Salvia miltiorrhiza* Bunge	Monomer	Sal B CF: C_36_H_30_O_16_	1.5, 3 mg/kg/d; 12.5–50 µM	8 weeks; 24 h	Positive metformin	In vivo In vitro	Mice Cells	Smad7↓, TGF‐β1↓	TGF‐β1 signaling pathway	[75]
		BB	Monomer	1‐DNJ CF: C_6_H_13_NO_4_	5.0, 10.0 mg/kg	6 weeks	Negative	In vivo	Mice	TGF‐β↓, N‐GlcNAc↓	TGF‐β/Smad2/3 pathway	[76]
		*Salvia miltiorrhiza* Bunge	Monomer	SAA CF: C_26_H_22_O_10_	10, 20 mg/kg; 25 μmol/L	4 weeks; 24 h	Negative	In vivo In vitro	Mice Cells	PKM2↓	Inhibition of PKM2 activity	[77]
		Paeonia lactiflora	Monomer	PF CF: C_23_H_28_O_11_	0.01 g/kg	6 weeks	Positive MH	In vivo In vitro	Rats Cells	PKCβ1↓	Reduction of PKCβ1	[78]
		ZGJTSXF	Herbal formula	Panax ginseng, Astragalus membranaceus, Rehmannia glutinosa, Pueraria lobata, Cornus officinalis, Salvia miltiorrhiza, Coptis Chinensis, *Ophiopogon japonicus*, Crataegus	33.67 g/kg/d	4 weeks	Negative	In vivo	Mice	TMAO↓	TMAO/PERK/FoxO1 signaling pathway	[79]
		Yangxinshi	Herbal formula	Ginseng, Rhizoma corydalis, Codonopsis pilosula, Pueraria lobata, hawthorn, and so on.	5 g/L	24 h	Negative	In vitro	Cells	Cbl‐b↑, p‐smad2↓, α‐SMA↓	Cbl‐b/smad2 pathway	[80]
		GN	Herbal formula	Ginseng, Atractylodes macrocephala, Poria cocos, yam, xylooligosaccharide	30 g/kg/d	15 days	Negative	In vivo In vitro	Mice Cells	TMBIM6↓		[81]
		HGWWD	Herbal formula	NA	60 g/kg/d	10 weeks	Negative	In vivo	Mice	Endothelial arginase 1↓	Arginase 1‐NO signaling	[82, p. 1]
		SSYX	Chinese patent medicine	Sodium danshensu, chlorogenic acid, paeoniflorin, spinosin, salvianolic acid B, berberine hydrochloride, ginsenoside Rb1, schisantherin A	200, 100, and 50 mg/kg/d	4 weeks	Negative	In vivo	Rats	Smad7↓, TGF‐β1↓, col‐1↑, col‐3↑	TGF‐β1/Smad signaling	[83]
		DHI	Chinese patent medicine	Radix Salviae Miltiorrhizae, Flos Carthami	1.3 mL/kg	35 days	Positive metformin	In vivo	Mice	VEGF‐A↑, VEGFR‐2↑, PPARδ↑, PPARγ↑	VEGF/VEGFR‐2 and PPARδ pathways	[84]

Cerebrovascular disease	Intracellular inflammatory pathway	Coptis chinensis	Monomer	Berberine CF: C_20_H_18_NO_4_	187.5 mg/kg/d	NA	Positive Met	In vivo	Rats	NF‐κB↓, TNF‐α↓, IL‐1↓, IL‐18↓,	Inhibitory inflammatory pathway	[85]
		*Salvia miltiorrhiza* Bunge	Chinese patent medicine	Salvianolate lyophilized	5.25, 10.5, or 21 mg/kg	14 days	Positive edaravone	In vivo	Rats	MMP9↓, COX‐2↓, TNF‐α↓, ICAM‐1↓	Inhibition of MMP and inflammatory factors	[86]
	Oxidative stress pathway	*Flos Puerariae*	Single medicinal botanical drug	*Flos Puerariae* extract	50, 100, or 200 mg/kg/d	10 weeks	Negative	In vivo	Rats	MDA↓, SOD↑, CAT↑, GSH‐Px↑	Ameliorated OS	[87]
	Apoptosis pathway	*Curcuma longa* L.	Monomer	Curcumin CF: C_21_H_20_O_6_	40 mg/kg	NA	Negative	In vivo	Rats	Bcl‐2↓	Antiapoptotic role	[88]
	Others	Coptis chinensis	Monomer	Berberine CF: C_20_H_18_NO_4_	1.0 g/kg/d	8 weeks	Negative	In vivo	Rats	miR‐133a↓	Suppression of miR‐133a ectopic expression	[89]

*Note:* SIRT1, sirtuin 1; AMPK, 5′‐adenosine monophosphate–activated protein kinase; PGC‐1α, proliferator‐activated receptor gamma coactivator 1‐alpha; SMYA, Si‐Miao‐Yong‐An decoction; GCGR, glucagon receptor; GreR, Glycyrrhizae Radix et Rhizoma; FTZ, Fufang‐Zhenzhu‐Tiaozhi capsule; CD36, cluster of differentiation 36; NLRP3, NLR family pyrin domain–containing 3; TSN, tanshinone IIA; IL‐6, interleukin 6; CHE, chelerythrine; CSE, cystathionine‐γ‐lyase; PKC, recombinant protein kinase C; APS, Astragalus polysaccharides; MMP‐2, matrix metalloproteinase‐2; IL 1β, interleukin 1 beta; Sch B, Schisandrin B; MyD88, myeloid differentiation factor88; LCME, *Lycium chinense* leaf extract; ICAM‐1, intercellular adhesion molecule‐1; MMPs, matrix metalloproteinases; IL‐18, interleukin 18; NR1, Notoginsenoside R1; IL‐10, interleukin 10; DOE, *Dendrobium officinale*; Myr, Myricitrin; PI3K, phosphoinositide 3‐kinase; AKT, protein kinase B; JLD, JinLiDa granules; TP53, tumor suppressor p53; NOX, nicotinamide adenine dinucleotide phosphate oxidase; Nrf2, nuclear factor erythroid 2–related factor 2; MDA, malondialdehyde; SOD, superoxide dismutase; GSH‐Px, glutathione peroxidase; ASI, asiaticoside; GSH, glutathione; OJP1, *O. japonicus* polysaccharide; Andro, Andrographolide; CAT, catalase; Cur, Curcumin; Bai, Baicalein; THJ, Taohuajing; YNJ, YuNü‐Jian; NQO1, recombinant NADH dehydrogenase, Quinone 1; WXKL, Wenxin Keli; LC3II, light chain 3 II; SJTYD, Shengjie Tongyu decoction; mTOR, mammalian target of rapamycin; LC3A‐II, microtubule‐associated protein 1 light chain 3‐II; HDC, Huangqi‐Danshen Metabolite; ZGJTSXF, Zuogui Jiangtang Shuxin formula; Bcl‐xL, B‐cell lymphoma‐extralarge; QSYQ, Qishen Yiqi drop pill; QGQXM, Qigui Qiangxin mixture; HJT, Hongjingtian injection; Sal B, salvianolic acid B; 1‐DNJ, 1‐deoxynojirimycin; N‐GlcNAc, N‐acetylglucosamine; TMAO, trimethylamine‐N‐oxide; p‐smad2, phospho‐Mothers Against Decapentaplegic Homolog 2; GN, Ginseng Dingzhi decoction; TMBIM6, transmembrane BAX inhibitor motif containing 6; SSYX, Shensong Yangxin capsule; Smad7, Recombinant Mothers Against Decapentaplegic Homolog 7; col‐1, collagen I; col‐3, collagen III; DHI, Danhong injection; PF, Paeoniflorin; PKCβ1, recombinant protein kinase C β1; Ad‐A1, adenoviral arginase 1; HGWWD, Huangqi Guizhi Wuwu decoction; COX‐2, cyclooxygenase 2; CC, AMPK inhibitor; RSG, rosiglitazone.

Abbreviations: α‐SMA, α‐smooth muscle actin; AED, aloe‐emodin derivative; ARP, *Anoectochilus roxburghii* polysaccharide; ASR, Angelicae Sinensis Radix; Bax, BCL2‐associated X; BB, Bombyx Batryticatus; Bcl‐2, B‐cell lymphoma‐2; Cbl‐b, Casitas B lymphoma‐b; CF, chemical formula; CPT1, carnitine palmitoyl transferase 1; FABP3, fatty acid binding protein 3; GSC, Ginseng‐Sanqi‐Chuanxiong; hs‐CRP, high‐sensitivity C‐reactive protein; LJF, Lonicerae Japonicae Flos; MCP‐1, monocyte chemoattractant protein‐1; MH, metformin hydrochloride; NA, not applicable; NF‐κB, nuclear factor‐kappa B; NO, nitric oxide; NOS, nitric oxide synthase; PKM2, pyruvate kinase M2; PNS, *Panax notoginseng* saponin; PPARα, peroxisome proliferator–activated receptor α; PPARδ, peroxisome proliferator–activated receptor δ; PPARγ, peroxisome proliferator–activated receptor γ; ROS, reactive oxygen species; SAA, salvianic acid A; sAT, saponins of Aralia taibaiensis; SFI, Shen‐fu injection; SMS, ShengMai‐San; SR, Scrophulariae Radix; TGFβ1, transforming growth factor β1; TNF‐α, tumor necrosis factor alpha; TwHF, Tripterygium wilfordii Hook F; VEGF‐A, vascular endothelial growth factor A; VEGFR‐2, VEGF receptor‐2.

Si‐Miao‐Yong‐An decoction (SMYA) is known for its lipid‐lowering properties and may offer therapeutic benefits in the management of DCM. The study explored the role of SMYA on the function of the heart in diabetic mice and its underlying molecular mechanisms [24]. In streptozotocin (STZ)‐induced diabetic mice, oral administration of SMYA significantly improved cardiac function, as evaluated by echocardiographic analysis. Cardiac histopathological alterations were evaluated using hematoxylin–eosin (H&E) staining, TUNEL assay (for apoptosis), and transmission electron microscopy (TEM) to assess ultrastructural changes. The protein expression levels of key components in the GLC/AMPK/NF‐κB and GLC/peroxisome proliferator–activated receptor α (PPARα)/PGC‐1α signaling pathways were quantitatively assessed using immunohistochemistry and Western blot analysis. The findings demonstrated that SMYA improved both systolic and diastolic cardiac function, and preserved the integrity of myofilament structure and mitochondrial function. Furthermore, SMYA reduced the expression levels of GLC receptor (GCGR), PGC‐1α, PPARα, and p‐NF‐κB, while increasing p‐AMPK in diabetic mice [90]. Collectively, SMYA ameliorates DCM in mice by modulating glucolipid metabolic homeostasis.

Fufang‐Zhenzhu‐Tiaozhi (FTZ) is a patented CHM metabolite recognized for its multifaceted effects in preventing and treating glycolipid metabolic disorders. Its therapeutic action is grounded in the traditional principles of “regulating the liver, activating pivot functions, and eliminating turbidity.” This study elucidates the regulatory mechanisms by which FTZ mitigates glycolipid metabolic dysregulation and mitochondrial dynamic imbalances in DCM, establishing a mechanistic foundation for its cardioprotective efficacy in diabetes [25]. The results showed that FTZ preserved cardiac function by downregulating proteins related to free fatty acid (FFA) uptake, including cluster of differentiation 36 (CD36), carnitine palmitoyl transferase 1 (CPT1), and fatty acid binding protein 3 (FABP3). In addition, FTZ regulated mitochondrial dynamics through suppressing fission and motivating fusion. It also restored the expression of key proteins related to glucolipid metabolism in palmitic acid–treated cardiomyocytes [91]. Therefore, FTZ improves cardiac function in the diabetic mice by reducing fasting blood glucose levels, preventing weight loss, correcting lipid metabolic disorders, and restoring mitochondrial dynamics.

#### 1.2.2. Intracellular Inflammatory Pathway

##### 1.2.2.1. Monomer

**FIGURE 2:**
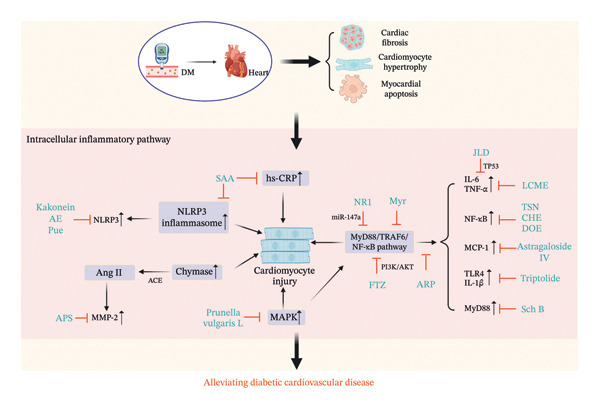
The mechanism of Chinese herbal medicine in treating diabetic cardiovascular diseases through the intracellular inflammatory pathway. DM, diabetic mellitus; NLRP3, NLR family pyrin domain–containing 3; TNF‐α. Tumor necrosis factor alpha; IL‐6, interleukin 6; NF‐κB, nuclear factor‐kappa B; hs‐CRP, high‐sensitivity C‐reactive protein; MCP‐1, monocyte chemoattractant protein‐1; TLR4, Toll‐like receptors 4; MyD88, myeloid differentiation factor 88; MMP‐2, matrix metalloproteinase 2; IL‐1β, interleukin 1‐beta; MAPK, mitogen‐activated protein kinases; SAA, salvianic acid A; ARP, *Anoectochilus roxburghii* polysaccharide; Pue, puerarin; NR1, notoginsenoside R1; TSN, tanshinone IIA; CHE, chelerythrine; LCME, *Lycium chinense* leaf extract; DOE, *Dendrobium officinale*; Myr, myricitrin; FTZ, Fufang‐Zhenzhu‐Tiaozhi capsule; APS, astragalus polysaccharides; Sal B, salvianolic acid B; JLD, JinLiDa granules.

Salvianolic acid A (SAA), as a principal bioactive constituent isolated from Salvia miltiorrhiza Bunge, exhibits potential therapeutic efficacy in the management of metabolic disorders attributable to its marked anti‐inflammatory activities. Given the pivotal role of chronic inflammation in the pathogenesis of Type 2 DM (T2DM) complicated by AS, SAA may confer distinct therapeutic advantages in AS management. This study investigated the effects of SAA on metabolic dysfunction in Zucker diabetic fatty (ZDF) rats induced by combined high‐fat diet (HFD) feeding and vitamin D3 injections [26]. Rats treated with a high dose of SAA showed reduced hemoglobin A1c (HbA1c) levels, although blood glucose levels remained unchanged. SAA improved lipid profiles by significantly reducing CHOL, LDL‐C, and triglyceride (TG). SAA exerted protective effects against early‐stage AS lesions in aortic tissues [92]. Furthermore, SAA significantly reduced serum high‐sensitivity C‐reactive protein (hs‐CRP) levels and inhibited both NLRP3 inflammasome and NF‐κB signaling pathway activation [93, 94] (Figure 2).

Tanshinone IIA (TSN), a major bioactive constituent of the tanshinone family, demonstrates extensive cardioprotective properties [95]. This study examined the effects of TSN on myocardial infarct (MI) size, cardiac function, inflammation, and apoptosis in diabetic rats undergoing MI/reperfusion (MI/R) [27]. Results indicated that TSN significantly decreased myocardial infarction area, enhanced left ventricular ejection fraction (LVEF), and reduced cardiomyocyte apoptosis relative to the untreated MI/R group. Western blot analysis demonstrated that TSN treatment significantly upregulated phosphorylation of protein kinase B (AKT) while concurrently downregulating NF‐κB phosphorylation in myocardial tissue. The cardioprotective effects of TSN were abolished by pretreatment with wortmannin, which resulted in increased MI size, reduced LVEF, elevated cardiomyocyte apoptosis, inhibited AKT phosphorylation, increased NF‐κB phosphorylation, and heightened production of inflammatory cytokines, such as TNF‐α and IL‐6 [96]. Overall, prophylactic administration of TSN attenuates MI area and enhances cardiac functional recovery after MI/R injury in diabetic rats, potentially mediated through its anti‐inflammatory and antiapoptotic properties.

Chelerythrine (CHE), a bioactive benzophenanthridine alkaloid, has been extensively characterized as a potent inhibitor of protein kinase C (PKC). This study systematically investigated the therapeutic potential of CHE in attenuating myocardial injury following renal I/R‐induced MI (RI/R‐MI) in STZ‐induced diabetic rat models [28]. Pretreatment with CHE improved cardiac function by reducing biomarkers of myocardial injury [97], decreasing oxidative stress, augmenting H_2_S levels, upregulating cystathionine‐γ‐lyase (CSE) expression, and downregulating NF‐κB, PKC‐α, and PKC‐β2. These findings suggest that CHE pretreatment exerts cardioprotective effects against RI/RMI, likely through activation of the endogenous CSE/H_2_S pathway via modulation of the PKC/NF‐κB signaling axis.

Kakonein, an isoflavone found in *Pueraria* DC., has been shown to effectively ameliorate diabetes and its associated complications. This study investigated whether kakonein alleviates cardiovascular endothelial dysfunction through inhibiting inflammation [29]. STZ‐induced hyperglycemia was achieved in male C57BL/6J mice through intraperitoneal injection. Immunofluorescence and ELISA assays confirmed the cardioprotective effects of kakonein on vascular endothelial junctions and on the NLRP3 inflammasome activation. The role of autophagy in regulating the NLRP3 inflammasome was further examined using immunofluorescence, Western blotting, and RT‐qPCR. The finding demonstrated that kakonein recovered endothelial function and suppressed NLRP3 inflammasome activation. Notably, kakonein reduced NLRP3 protein expression without affecting the transcription of caspase‐1 and NLRP3 [98, 99]. Additionally, kakonein inhibited hyperglycemia‐induced vascular endothelial dysfunction and NLRP3 inflammasome activation, producing effects similar to those of an autophagy agonist. Overall, these findings demonstrate that kakonein protects against hyperglycemia‐induced cardiac vascular dysfunction by modulating NLRP3 inflammasome activity through an autophagy‐dependent mechanism.

Astragalus polysaccharide (APS) is a class of water‐soluble polysaccharide compounds extracted and isolated from the Astragalus membranaceus (Huangqi), serving as one of its primary bioactive constituents. APS, commonly utilized in CHM for their immune‐boosting properties, was examined in this study for their impact on cardiac function, cardiac ultrastructure, myocardial collagen deposition, matrix metalloproteinase (MMP) activity, glycosylated serum protein levels, myocardial enzymes, and the expression level of angiotensin II (Ang II), chymase, and ACE in diabetic hamster hearts [30]. DM was induced in the animals via intraperitoneal injection of STZ. Compared to conventional insulin therapy, APS demonstrated superior cardioprotective effects through significant reductions in myocardial collagen I and III deposition, inhibition of MMP‐2 activity, suppression of Ang II production, and downregulation of both chymase expression and p‐ERK1/2 phosphorylation [100]. However, APS administration did not significantly alter ACE protein levels or total Ang II expression. These findings indicate that APS therapy improves key indicators of DCM in hamsters, likely by inhibiting the chymase–Ang II axis and mitigating pathological cardiac remodeling.

Ferulic acid, derived from *Angelica sinensis*, and astragaloside IV, isolated from *Phelipanche astragali* (Mouterde) Holub, have demonstrated potential cardioprotective and antihyperglycemic properties. This study examined their combined cardioprotective effects and underlying mechanisms in mitigating cardiovascular endothelial dysfunction in STZ‐induced diabetic rats [31]. The combination of ferulic acid and astragaloside IV improved aortic endothelial integrity and significantly reduced HbA1c, TG, total CHOL (total TC), LDL‐C, and ox‐LDL levels. It also enhanced nitric oxide (NO) and endothelial NO synthase (eNOS) expression while suppressing NF‐κB P65, monocyte chemoattractant protein‐1 (MCP‐1), and TNF‐α activation [101], without causing adverse effects on liver or kidney function. In summary, the combined treatment effectively protected diabetic rats from cardiovascular endothelial dysfunction via NF‐κB pathway modulation, characterized by reduced ox‐LDL levels, increased NO and eNOS production, and downregulation of NF‐κB p65, TNF‐α, and MCP‐1.

Triptolide, a major active metabolite derived from the CHM *Tripterygium* Hook.f., has been investigated for its protective effects against DCM. This study explored how triptolide modulates the immune system and attenuates inflammation, thereby reducing cardiac fibrosis and improving LV function [32]. In diabetic rats, upregulation of NF‐κB p65 and Toll‐like receptors 4 (TLR4) was associated with elevated proinflammatory cytokines, increased CF, and impaired ventricular function. Notably, triptolide treatment improved both the structural integrity and functional performance of the LV. Triptolide significantly suppressed key immune mediators, including TLR4, NF‐κB p65, interleukin‐1β (IL‐1β), TNF‐α, CD68^+^ cells, MCP‐1, and vascular cell adhesion molecule‐1 (VCAM‐1) [102]. Additionally, triptolide reduced markers of CF, such as α‐smooth muscle actin (α‐SMA), vimentin, transforming growth factor‐β1 (TGF‐β1), and collagen aggregation. Overall, these findings suggest that triptolide exerts cardioprotective effects against DCM by suppressing the NF‐κB/IL‐1β and NF‐κB/TNF‐α/VCAM‐1 inflammatory pathways, as well as by downregulating the TGF‐β1/α‐SMA/vimentin fibrosis signaling axis [33].

Derived from Schisandra chinensis fruits, Schisandrin B (Sch B) represents a predominant dibenzocyclooctadiene derivative with significant biological activity. Sch B exerts potent anti‐inflammatory effects through multitarget mechanisms, significantly suppressing key proinflammatory cytokines (TNF‐α, IL‐6) [103]. Recent research has investigated the effects of Sch B, an anti‐inflammatory active metabolite, in the context of DCM [34]. SchB can effectively suppress hyperglycemia‐induced cardiomyocyte hypertrophy and fibrosis. Integrated transcriptomic and qPCR analyses demonstrate that Sch B selectively inhibits the myeloid differentiation factor88 (MyD88)–dependent inflammatory axis in high‐level glucose (HG) cardiomyocytes. Subsequent mechanistic investigations demonstrate that Sch B directly interacts with and suppresses MyD88 activation, while exhibiting no observable interference with MyD88‐independent TLR signaling pathways [104]. Additionally, Sch B exerts comprehensive cardioprotective effects in diabetic mice, preserving cardiac function, mitigating myocardial injury, and suppressing proinflammatory cytokine release. These findings indicate that the MyD88 in cardiomyocytes is a causative factor in DCM and that SchB specifically targets MyD88 to alleviate inflammation‐associated DCM.

Aloe emodin (AE), chemically defined as 1,8‐dihydroxy‐3‐(hydroxymethyl) anthraquinone, is an anthraquinone derivative isolated from aloe plant exudates. AE demonstrates significant cytoprotective effects against HG‐induced β‐cell glucotoxicity, manifested by marked suppression of proinflammatory mediators, including IL‐1β [105]. This research introduces a new type of metabolite, an aloe‐emodin derivative (AED), synthesized from the AE family [35]. AED is generated by covalently linking monomethyl succinate to the anthraquinone backbone of AE. The study investigated the therapeutic effects of AED in DCM. DM was experimentally induced in male Sprague Dawley rats through a combination of HFD feeding and STZ administration. Notable improvements in cardiac function were observed in the DCM rats treated with AED or AE over 4 weeks, with AED demonstrating superior therapeutic efficacy. AED attenuated inflammation by inhibiting the NLRP3 inflammasome‐mediated pyroptosis pathway [106]. KEGG pathway enrichment analysis demonstrated significant activation of the NOD‐like receptor signaling pathway in the intervention group relative to hyperglycemic controls. Moreover, NLRP3 overexpression abolished the antipyroptotic effects of AED in hyperglycemia‐treated H9C2 cells.

*Lycium chinense* Mill. (Solanaceae) is a renowned CHM with dual applications in both food and medicine. *Lycium chinense* leaf extract (LCME) exhibits a broad spectrum of pharmacological activities, encompassing antihyperlipidemic, antioxidant, antimicrobial, antiobesity, and anti‐inflammatory properties [107]. The research examined the roles of LCME in the fructose‐ and STZ‐induced diabetic rats [36]. Diabetic rats received oral LCME treatment for 5 weeks. The results showed elevated blood glucose levels; increased serum cardiac markers—specifically troponin T (TnT), creatine kinase‐MB (CK‐MB), lactate dehydrogenase (LDH), and aspartate aminotransferase (AST); and higher lipid profiles (TG and CHOL) in diabetic rats. Cardiac tissues exhibited increased expression of IL‐6, IL‐1β, TNF‐α, NF‐κB, malondialdehyde (MDA), and caspase‐3, along with reduced activities of superoxide dismutase (SOD), catalase (CAT), glutathione peroxidase (GPx), and glutathione (GSH) [108, 109]. LCME administration significantly reduced hyperglycemia and lowered serum levels of cardiac markers and lipids [36]. In addition, LCME effectively decreased oxidative and inflammatory mediators while enhancing antioxidant defenses in cardiac tissue [36, 110].

Pue, the major bioactive metabolite of *Pueraria lobata* (Willd.) Ohwi, is recognized for its potent anti‐inflammatory properties; however, its role in vascular protection remains less clearly defined. The research investigated the protective efficacy of Pue against diabetes‐induced vascular injury [37]. Pue diminished lipopolysaccharide–adenosine triphosphate (LPS–ATP) or HG‐induced cytotoxicity by suppressing ROS‐mediated NLRP3 inflammasome activation in the human umbilical vein endothelial cells (HUVECs). This was evidenced by reduced levels of ROS, nicotinamide adenine dinucleotide phosphate oxidase 4 (NOX4), and caspase‐1 activity, as well as decreased expression of NLRP3, GSDMD, cleaved caspase‐1, interleukin‐18 (IL‐18), and IL‐1β [111]. The ROS inducer CoCI_2_ diminished Pue’s protective effects against LPS–ATP‐induced pyroptosis [112]. In addition, NLRP3 knockdown enhanced the inhibitory effect of Pue on pyroptosis, whereas NLRP3 overexpression counteracted it. Pue also reduced ROS levels and NLRP3 inflammasome‐related protein expression in the aortas of diabetic rats. The findings indicate that Pue mitigates LPS–ATP‐ or HG‐induced endothelial injury by inhibiting the ROS–NLRP3 signaling pathway.

Notoginsenoside R1 (NR1), a key bioactive metabolite of *Panax quinquefolius* L., has been shown to counteract HG‐induced endothelial dysfunction. In this study, HG‐treated HUVECs were subsequently exposed to NR1 [38]. Apoptosis was evaluated using flow cytometry, and angiogenic capacity was assessed through tube formation assays. The relative expression of miR‐147a, serum concentrations of inflammatory cytokines (TNF‐α, IL‐6, IL‐10), and oxidative stress parameters (MDA levels, SOD, and GPx activities) were quantitatively assessed. The protein expression levels of the MyD88/TRAF6/NF‐κB signaling axis, along with apoptosis‐related markers (Bcl‐2, Bax, and Caspase‐3), were quantified using Western blot analysis. The findings indicated that NR1 reduced apoptosis and enhanced tube formation in HG‐treated HUVECs. Moreover, NR1 attenuated oxidative stress and inflammation by suppressing HG‐induced activation of the MyD88/TRAF6/NF‐κB pathway [113]. NR1 also upregulated miR‐147a, which directly targets MyD88. Upregulation of miR‐147a suppressed the MyD88/TRAF6/NF‐κB signaling pathway, while miR‐147a inhibition abolished the cytoprotective effects of NR1 by restoring pathway activity [38, 114, 115].

Myricitrin (Myr), a bioactive flavone glycoside, is predominantly found in the root bark of several medicinal plants, such as Myrica cerifera, Myrica esculenta, and Ampelopsis grossedentata. Myr has been demonstrated to exhibit potent anti‐inflammatory and antifibrotic activities in preclinical studies [116]. This research evaluated the effects of Myr on cardiac function in diabetic mice induced by STZ and in H9C2 cardiomyocytes exposed to advanced glycation end products (AGEs) [39]. In vitro, Myr pretreatment markedly reduced AGEs‐induced inflammatory cytokine production, decreased ROS accumulation, and attenuated apoptosis, hypertrophy, and fibrosis in cardiomyocytes. These effects were linked to the activation of nuclear factor erythroid 2–related factor 2 (Nrf2) and the suppression of NF‐κB signaling. In vivo, Myr significantly decreased levels of cardiomyopathy‐related enzymes, inflammatory cytokines, and apoptotic proteins, while enhancing diastolic function and alleviating myocardial damage. Mechanistically, Myr is thought to reverse HG‐induced Nrf2 inhibition by modulating ERK phosphorylation and AKT signaling in the diabetic heart [117]. Overall, these findings demonstrate that Myr provides cardioprotective effects in DCM through inhibiting inflammation, oxidative stress, and apoptosis.

#### 1.2.3. Single Medicinal Botanical Drug

Prunella vulgaris L., originally documented in Shen Nong’s Herbal Classic, is a medicinal plant containing pharmacologically active compounds including triterpenes, sterols, flavonoids, and organic acids. Scientific studies have validated its significant hypotensive and hypoglycemic properties. This research sought to explore the bioactive metabolites, potential targets, and signaling pathways of Prunella vulgaris L. to examine its “multitarget, multipathway” molecular mechanisms for DM complicated by hypertension [40]. Through network pharmacology analyses, 11 active metabolites, 41 pivotal targets, and 16 signaling pathways were screened. The primary active metabolites effective against DM complicated by hypertension were quercetin and kaempferol, with potential targets including insulin and IL‐6. Key signaling pathways involved included AGE receptor for AGE (RAGE), mitogen‐activated protein kinases (MAPK), TNF, and PI3K–AKT pathways [118]. These signaling pathways modulate critical biological processes, including apoptosis and inflammatory cascade activation.

*Anoectochilus roxburghii* (Wall.) Lindl. has been utilized in China as a traditional remedy for diabetes. Its primary bioactive metabolite, *Anoectochilus roxburghii* polysaccharide (ARP), has been investigated for its vascular protective effects [41]. ARP significantly reduced blood glucose levels. Histological analyses revealed that ARP enhanced vascular repair. In vitro, ARP pretreatment markedly inhibited the production of ROS, ICAM‐1, and MCP‐1 in the HG‐induced HUVECs [119]. ARP also suppressed HG‐induced MMP activity by upregulating tissue inhibitor of MMP (TIMP) expression, thereby maintaining vascular structure balance. Additionally, pretreatment with ARP markedly downregulated the p‐p38 MAPK and NF‐κB p65 in HUVECs. In conclusion, the cardiovascular protective effects of ARP may be mediated through the p38 MAPK and NF‐κB pathways.

*Dendrobium officinale* extract (DOE) is a bioactive mixture derived from Dendrobium species (Orchidaceae) through aqueous/alcoholic/supercritical extraction, containing polysaccharides, alkaloids, flavonoids, and phenolics. *Dendrobium officinale* Kimura & Migo, a pharmacopeial CHM, has been extensively utilized in traditional medical systems across China and Southeast Asia for centuries. A recent preclinical study evaluated the impact of DOEs on DCM in mice [42]. Diabetes was induced by intraperitoneal STZ injections administered over five consecutive days. Successful induction of diabetes was confirmed by significant elevations in CK and LDH levels in diabetic mice. DOE treatment reduced the heart‐to‐body weight ratio (HW/BW) and exhibited notable hypoglycemic activity. Additionally, DOE significantly decreased serum levels of CK, LDH, TC, and TG, while reducing MDA production and enhancing total superoxide dismutase (T‐SOD) activity. Histological assessments using Oil Red O and Sirius Red staining demonstrated marked improvements in cardiac injury, including reduced lipid accumulation and attenuated collagen deposition following DOE administration. Furthermore, Western blot analysis demonstrated that DOE markedly downregulated the expression of TGF‐β, fibronectin, collagen‐1, NF‐κB, IL‐1β, and TNF‐α, indicating suppression of inflammatory and fibrotic pathways [120].

##### 1.2.3.1. Herbal Formula

The patented FTZ formula integrates eight medicinal plants—notably *Coptis chinensis*, *Salvia miltiorrhiza*, and *Panax notoginseng*—with demonstrated lipid‐modulating properties. This study aimed to establish a practical minipig model of DM complicated by coronary heart disease (DM‐CHD) and to evaluate the cardioprotective effects of FTZ [43]. This DM‐CHD model was successfully induced in minipigs presenting with glycolipid metabolic disorders, coronary artery thickening, and myocardial injury. FTZ treatment significantly attenuated coronary artery thickening and preserved myocardial structure. Moreover, FTZ treatment significantly attenuated proinflammatory cytokine release while concurrently upregulating key PI3K/AKT pathway effectors in myocardial tissue [121]. In conclusion, FTZ confers cardioprotection in DM‐CHD by attenuating inflammatory responses and modulating the PI3K/AKT signaling axis.

##### 1.2.3.2. Chinese Patent Medicine

JinLiDa granules (JLD) are a patented Chinese herbal formulation comprising 17 medicinal ingredients, primarily exhibiting spleen‐strengthening (*Jian-Pi*), fluid metabolism regulation (*Yun-Hua Jin-Ye*), qi‐tonifying (*Yi-Qi*), and yin‐nourishing (*Zi-Yin*) pharmacological activities. JLD is a CHM commonly prescribed for T2DM characterized by Qi and Yin deficiency. Although previous studies have indicated that JLD may exert protective effects against DCM, the underlying mechanisms remain insufficiently defined. This research integrates network pharmacology prediction with experimental validation to systematically decipher the mechanistic foundation of JLD’s therapeutic actions [44]. The analysis identified tumor suppressor p53 (TP53) as a key regulatory target implicated in the inflammatory and fibrotic processes associated with DCM. Experimental results demonstrated that JLD administration ameliorated ventricular wall thickening, attenuated cardiac hypertrophy, and reduced the expression of inflammation‐related factors in cardiomyocytes. Morphological improvements in cardiomyocytes were also observed following treatment. Notably, TP53 expression, along with the TNF and TGF‐β1 signaling pathways, was significantly modulated by JLD. TP53 inhibition suppressed the activation of these pathways under HG conditions [122], whereas TP53 overexpression promoted their activation. In summary, JLD exerts cardioprotective effects in DCM primarily by regulating TP53, thereby modulating the TNF and TGF‐β1 signaling pathways and ultimately mitigating cardiomyocyte hypertrophy.

#### 1.2.4. Oxidative Stress Pathway

##### 1.2.4.1. Monomer

**FIGURE 3:**
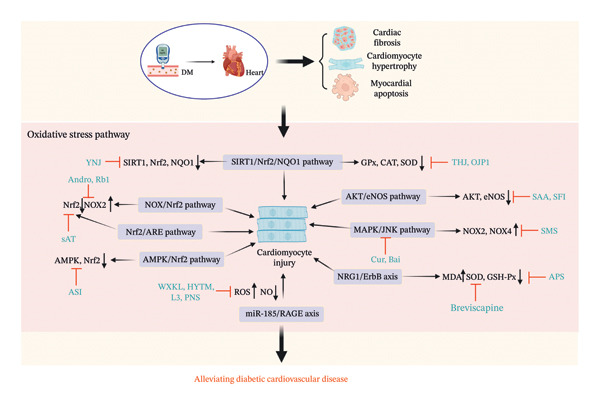
The mechanism of Chinese herbal medicine in treating diabetic cardiovascular diseases through the oxidative stress pathway. DM, diabetic mellitus; SIRT1, sirtuin 1; Nrf2, nuclear factor erythroid 2–related factor 2; NQO1, recombinant NADH dehydrogenase, Quinone 1; NOX, nicotinamide adenine dinucleotide phosphate oxidase; AMPK, 5′‐adenosine monophosphate–activated protein kinase; AKT, protein kinase B; eNOS, endothelial nitric oxide synthase; NOX2, nicotinamide adenine dinucleotide phosphate oxidase 2; NOX4, nicotinamide adenine dinucleotide phosphate oxidase 4; MDA, malondialdehyde; SOD superoxide dismutase; GSH‐Px, glutathione peroxidase; ROS, reactive oxygen species; CAT, catalase; MAPK, mitogen‐activated protein kinases; ASI, asiaticoside; Andro, Andrographolide; OJP1, *O. japonicus* polysaccharide; PNS, *Panax notoginseng* saponin; sAT, saponins of Aralia taibaiensis; Cur, curcumin; Bai, baicalein; THJ, Taohuajing; YNJ, YuNü‐Jian; SMS, ShengMai‐San; HYTM, Huayu Tongmai granules; WXKL, Wenxin Keli; SFI, Shen‐fu injection; SAA, salvianic acid A; APS, astragalus polysaccharides.

Andrographolide (Andro), a key metabolite isolated from *Andrographis paniculata* (Burm.f.) Wall. ex Nees, exhibits significant antioxidant and cytoprotective properties. This study investigated the therapeutic effects of Andro on STZ‐induced DCM in mice, with a focus on its cardioprotective mechanisms [45]. Andro treatment exhibited a dose‐dependent amelioration of oxidative stress, suppression of cardiomyocyte apoptosis, and significant improvements in cardiac function and pathological hypertrophy. Additionally, Andro inhibited hyperglycemia‐induced ROS production by suppressing NOX and enhancing Nrf2 expression. These data demonstrate that Andro’s cardioprotective effects are mediated through modulation of the NOX/Nrf2 pathway [123] (Figure 3).

Salvianic acid A (SAA), a bioactive metabolite isolated from Salvia miltiorrhiza Bunge, has been extensively utilized in the management of cardiovascular disorders. The research examined the protective effects of SAA on rat endothelial progenitor cells (EPCs) cultured under HG conditions [46]. The EPCs exhibited typical cobblestone morphology and were positive for FITC‐UEA‐1 and Dil‐ac‐LDL staining. Although HG exposure reduced cell activity, treatment with SAA at various concentrations did not significantly alter viability under these conditions. However, SAA attenuated apoptosis and LDH release, while promoting tube formation, improving cellular activity, and enhancing NO production [124]. In addition, SAA increased the ratios of p‐AKT to total AKT and p‐eNOS to total eNOS in EPCs exposed to HG. The PI3K inhibitor LY294002 effectively blocked the protective effects of SAA. Collectively, these findings indicate that SAA protects EPCs against HG‐induced dysfunction through activation of the AKT/eNOS signaling pathway.

Breviscapine is an efficient scavenger of oxygen free radicals and helps preserve redox homeostasis, thereby protecting cells from oxidative injury [47]. This research examined the impact of breviscapine on ICAM‐1 expression, ATPase activities, and oxidative stress in the I/R myocardium of diabetic rats [47]. This finding revealed that breviscapine markedly increased the activities of Na^+^‐K^+^‐ATPase, Ca^2+^‐ATPase, and Mg^2+^‐ATPase in the diabetic rats compared with untreated controls. Although MDA levels were elevated in diabetic controls, breviscapine significantly reduced MDA concentrations in both diabetic and nondiabetic animals. Furthermore, the activities of SOD and GSH‐Px, which were significantly reduced in diabetic control groups, were markedly restored following breviscapine treatment. ICAM‐1 expression was also upregulated in diabetic controls but was suppressed by breviscapine in both groups [125]. Overall, these findings suggest that breviscapine mitigates myocardial I/R injury in diabetic rats by decreasing oxidative stress, downregulating ICAM‐1 expression, and enhancing mitochondrial ATPase activities.

APS, a major bioactive component derived from Astragalus mongholicus Bunge, possesses notable antioxidative capabilities. Previous studies have shown that APS can activate the ERK1/2 signaling cascade, thereby promoting myocardial collagen accumulation and enhancing cardiac performance [126]. In DCM, dysfunction of the NRG1/ErbB axis has been identified, highlighting its physiological significance [127]. In the AGE‐induced DCM model used in this study, APS enhanced cardiomyocyte proliferation and mitigated apoptosis. It also reduced intracellular ROS production, boosted the activities of SOD and GSH‐Px, and lowered MDA and NO levels, confirming its antioxidative action [128]. Western blot results further revealed that APS upregulates NRG1 and facilitates ErbB2/4 phosphorylation. Elevated NRG1 subsequently activates downstream PI3K and AKT phosphorylation, indicating that the protective influence of APS is mediated via the NRG1/ErbB pathway and its associated PI3K/AKT signaling cascade [48, 127]. Moreover, the ErbB inhibitor canertinib partially counteracted APS‐induced improvements in cell survival, oxidative stress, and pathway activation. Collectively, these findings support the notion that APS alleviates DCM primarily through modulation of the NRG1/ErbB signaling network.

Asiaticoside (ASI) and activation of the AMPK/Nrf2 signaling pathway are known to exert cardioprotective effects; however, their specific roles in DCM remain insufficiently defined. In this study, DCM was induced in mice via intraperitoneal STZ injections combined with an HFD [49]. The mice were subsequently treated with ASI, along with pharmacological inhibitors of AMPK and Nrf2. Cardiac function, oxidative stress, and autophagy were systematically evaluated. DCM mice showed elevated oxidative stress and impaired autophagy. ASI administration activated the AMPK/Nrf2 pathway, leading to improvements in these pathological changes. Notably, the cardioprotective effects of ASI were abolished by AMPK and Nrf2 inhibitors, such as compound C and ML‐385 [129]. Overall, these findings indicate that ASI mitigates DCM progression primarily through activation of the AMPK/Nrf2 signaling axis.

L3, a curcumin analog derived from *Curcuma longa* L., is under investigation for its therapeutic potential in diabetic atherosclerosis and the mechanisms. This research assessed the impacts of L3 on glucolipid metabolism, antioxidant capacity, AS‐related markers, and organ pathology in a diabetic mouse model induced by STZ and an HFD [50]. The findings demonstrated that L3 treatment improved glucolipid metabolism, reduced oxidative stress, enhanced antioxidant enzyme activities, and increased NO levels in both plasma and the aortic arch [130]. Additionally, L3 decreased ROS production and downregulated oxidized LDL receptor‐1 expression, thereby alleviating lipid deposition and atherosclerotic lesions. These results indicate that L3 may help prevent diabetes and its associated complications by mitigating oxidative stress.

*Ophiopogon japonicus* (Thunb.) Ker Gawl., a CHM, has long been used to manage cardiovascular and chronic inflammatory disorders. This study examined the impact of *O. japonicus* polysaccharide (OJP1) on the cardiovascular injury in the diabetic rats [51]. The results revealed that OJP1 observably reduced MDA levels and enhanced the activities of GPx, CAT, and SOD in the diabetic rat heart. Furthermore, OJP1 treatment decreased levels of AGE, soluble ICAM‐1 (sICAM‐1), hs‐CRP, NO, and endothelin‐1. It also lowered endothelin‐1 mRNA levels while increasing eNOS mRNA levels. The results of histopathological analysis indicated that the OJP1 mitigated cardiac damage in the diabetic rats [131]. Collectively, these findings suggest that OJP1 strengthens endogenous antioxidant defenses and ameliorates diabetes‐related cardiovascular dysfunction.

Ginsenoside Rb1 demonstrated a dual effect by suppressing the activity of both the Kelch‐like ECH‐associated protein 1 (Keap1) and p47^phox^ in luciferase reporter assays. In the ECs, Rb1 treatment boosted cell activity while reducing inflammation, oxidative stress, and apoptosis, as well as improving mitochondrial quality under ox‐LDL and HG conditions. Rb1 directly interacted with Keap1, promoting its ubiquitination and proteasomal degradation via lysine residues through the recruitment of E3 ligase synovial apoptosis inhibitor 1 (SYVN1) [52]. This interaction led to the release of Nrf2 from Keap1, allowing Nrf2 to translocate to the nucleus and form a complex with PGC‐1α. Additionally, Rb1 bound to p47^phox^, inhibiting its phosphorylation and membrane translocation, thereby preventing the assembly of the NOX2 complex [132]. Notably, Rb1’s stabilization of cytoplasmic p47^phox^ further potentiated Nrf2 activation. Furthermore, Rb1 reduced atherosclerotic plaque, oxidative stress, and inflammation in the STZ‐induced ApoE^−/−^ mice, but these effects were abolished in ApoE^−/−^ mice lacking Nrf2 and PGC‐1α.

Panax notoginseng total saponins (PNS), the primary active metabolite in *Panax quinquefolius* L., are employed to treat diabetes. Nevertheless, the impact of PNS in DCM is unclear. In a study involving diabetic db/db mice, PNS was administered to assess its effects on lipid accumulation and cardiac function, along with the underlying mechanisms [53]. The results indicated that PNS observably lowered body fat content and ameliorated serum lipid profiles and antioxidant functions. Furthermore, PNS treatment alleviated lipid deposition in both adipose tissue and cardiac muscle, while enhancing both cardiac function and mitochondrial structure. In the H9C2 cells exposed to palmitic acid, PNS pretreatment significantly attenuated lipid accumulation and mitochondrial ROS levels, while improving ΔΨm and oxygen consumption rate [133]. Additionally, PNS administration enhanced the expression levels of proteins and genes related to glucolipid metabolism, antioxidant activity, and mitochondrial dynamics. Overall, PNS administration alleviated diabetes‐induced cardiac dysfunction through dual mechanisms: attenuating myocardial lipid accumulation and restoring redox homeostasis.

*Aralia taibaiensis* Z. Z. Wang & H. C. Zheng (sAT) is a clinically employed botanical agent for diabetes management, demonstrating significant free radical scavenging capacity and potent inhibitory effects on lipid peroxidation. A recent study aimed to determine whether sAT confers cardioprotective benefits in the context of diabetes [54]. Oxidative stress was induced in H9C2 cardiomyocytes using HG and glucose oxidase, and the protective effects of sAT were subsequently assessed. Exposure to HG and glucose oxidase markedly increased intracellular ROS production and oxidative damage, both of which were significantly attenuated by sAT treatment [134]. Further mechanistic investigation demonstrated that sAT treatment significantly enhanced nuclear accumulation of Nrf2 and transcriptionally activated its downstream antioxidant response elements. Importantly, the cytoprotective effects of sAT were substantially diminished following Nrf2 knockdown by siRNA. Collectively, these findings indicate that the Nrf2/ARE signaling pathway mediates the protective actions of sAT against hyperglycemia‐induced oxidative stress, supporting its therapeutic potential in DCM.

##### 1.2.4.2. Herbal Formula

Curcumin (Cur) has been demonstrated to mitigate oxidative stress by reducing ROS and superoxide generation, accompanied by enhanced heme oxygenase‐1 (HO‐1) activity [135]. Baicalein (Bai), meanwhile, exerts antioxidant effects primarily through the activation of the Nrf2‐dependent signaling pathway [136]. A recent study investigated the combined influence of Curcuma longa L.–derived Cur and Bai on vascular function [55]. The findings indicated that coadministration of Cur and Bai produced a synergistic protective effect on endothelial cells, leading to more pronounced reductions in fasting blood glucose and serum lipid levels in rats than either compound administered alone. In addition, the combination therapy helped preserve vascular structural integrity. Network pharmacology predicted that the Nrf2 and MAPK/JNK pathways were key contributors to the multitargeted actions of Cur and Bai, a prediction further supported by experimental validation [137, 138]. Overall, Cur and Bai jointly enhanced vascular endothelial resilience to oxidative stress, providing more robust protection against diabetic vascular injury compared with monotherapy.

Taohuajing (THJ), a formulation composed of *Prunus persica* (L.) Batsch, *Polygonatum sibiricum* Redouté, and *Carthamus tinctorius* L., has shown potential therapeutic value in DCM. A recent research investigated the cardioprotective efficacy of THJ in a DCM model and explored its underlying mechanisms [56]. T2DM was induced in C57BL/6 mice, which subsequently developed cardiac dysfunction, metabolic abnormalities, and myocardial fibrosis—hallmarks of DCM. THJ administration significantly ameliorated all these pathological changes. Moreover, THJ markedly attenuated oxidative stress by decreasing ROS and MDA levels while enhancing the enzymatic activities of GSH‐Px and SOD. It also reduced proinflammatory cytokine production and suppressed NLRP3 inflammasome activation [139]. Importantly, these beneficial effects were abolished by sirtinol, a SIRT1 inhibitor. Collectively, the findings suggest that THJ protects the myocardium from HG‐induced oxidative stress and inflammation via an SIRT1‐dependent mechanism that enhances endogenous antioxidant defenses.

YuNü‐Jian (YNJ) is recognized for its hypoglycemic activity and cardioprotective effects. The research explored the mechanisms by which YNJ acts against DCM [57]. Network pharmacology analysis was employed to elucidate the potential pathways and molecular targets of YNJ relevant to DCM. Subsequently, molecular docking analysis was conducted to evaluate the binding interactions between YNJ’s active metabolites and key target proteins, followed by visualization using AutoDock Vina and PyMOL [140]. The diabetes model was then used to validate these critical targets following YNJ treatment. Analysis of the PPI network identified several hub genes—Nrf2, SIRT1, MYC, and NAD(P)H: quinone oxidoreductase 1 (NQO1)—through topology analysis. Bioinformatics analyses suggested that these predicted targets were primarily associated with oxidative stress responses [141]. Molecular docking further demonstrated strong binding affinities between the core targets and YNJ’s active metabolites. In vivo, YNJ treatment significantly reduced cardiac fibrosis and collagen deposition, while upregulating the protein expression of SIRT1, Nrf2, and NQO1 in DCM mice. Taken together, these data demonstrate that YNJ mitigates DCM by specifically activating the SIRT1/Nrf2/NQO1 pathway.

SMS is traditionally used to treat ischemic cardio‐cerebrovascular diseases, and recent studies have highlighted its therapeutic potential in diabetic models. This study investigated whether SMS benefits DCM by reducing NOX‐mediated oxidative stress [58]. The findings indicated that diabetes resulted in elevated myocardial collagen deposition and fibrosis. Oxidative stress in diabetic cardiovascular injury was characterized by reduced total antioxidant capacity, elevated levels of 8‐iso‐prostaglandin‐F2α and 8‐hydroxy‐2ʹ‐deoxyguanosine, AMPKα inactivation, and upregulated expression of NOX2 and NOX4, accompanied by the translocation of NOX isoforms p67phox and p47phox. After 10 weeks of SMS treatment, diabetes‐induced myocardial structural abnormalities and apoptosis were significantly improved. SMS also reduced oxidative stress biomarkers in cardiac tissue [59]. Further mechanistic analysis demonstrated that SMS reversed the protein expression levels of NOX2 and NOX4 and prevented the translocation of NOX isoforms p47^phox^ and p67^phox^. Moreover, SMS motivated AMPK activation in the cardiac tissue of diabetic rats.

Huayu Tongmai granules (HYTM) are known for their therapeutic efficacy in diabetic angiopathy. The research systematically investigates the molecular mechanisms through which HYTM confers protection against diabetes‐induced vascular injury [60]. A diabetic rat model and an in vitro hyperglycemia‐induced endothelial cell model were established to investigate these effects. These findings demonstrated that HYTM significantly reduced blood glucose levels and intracellular ROS, while simultaneously enhancing NO production. In diabetic rats, miR‐185 expression was markedly downregulated [142]; however, HYTM treatment effectively restored its expression. Further analyses revealed that miR‐185 overexpression suppressed the expression of the RAGE, thereby reducing endothelial cell apoptosis. Overall, the findings suggest that HYTM exerts vasoprotective effects in diabetic angiopathy, at least in part, through modulation of the miR‐185/RAGE signaling axis.

##### 1.2.4.3. Chinese Patent Medicine

Wenxin Keli (WXKL), a CHM known for its antiarrhythmic properties, has been demonstrated to protect against cardiac arrhythmias by modulating ion channels. The research aimed to test the hypothesis that WXKL can enhance atrial remodeling in the diabetic rats by recovering mitochondrial function [61]. Various assessments were conducted, including echocardiography, hemodynamic evaluation, histology, electrophysiology studies, mitochondrial respiratory function assays, and western blots. In primary atrial fibroblasts, H_2_O_2_ treatment increased ROS levels, decreased MMP, and reduced oxygen consumption; these effects were reversed by WXKL [143]. In diabetic rats, WXKL treatment could result in a decrease in atrial fibrosis, an increase in left atrial diameter, improved atrial conduction velocity, decreased conduction heterogeneity, reduced atrial fibrillation inducibility, and enhanced mitochondrial protein expression. Thus, WXKL appears to improve atrial remodeling by modulating mitochondrial function and decreasing mitochondrial ROS levels in the diabetic rats.

Shen‐fu injection (SFI), a standardized traditional Chinese medicine extract derived from the roots of Panax ginseng and Aconitum carmichaelii, has demonstrated potent cardioprotective properties in preclinical models, as evidenced by its ability to mitigate myocardial ischemia–reperfusion injury and improve cardiac function. The research investigates whether SFI can attenuate MI/R injury in diabetes [62]. Diabetic rats induced by STZ were randomly distributed to four groups: Sham, I/R, SFI preprocessing, and SFI combined with wortmannin. With the exception of the Sham‐operated controls, all animals were subjected to 30 min of coronary artery occlusion, followed by 120 min of reperfusion to establish I/R injury. Compared with Sham‐operated controls, the MI/R group demonstrated significantly larger infarct size and elevated cardiomyocyte apoptosis rate. SFI pretreatment markedly reduced infarct size, caspase‐3 expression, and myocardial MDA levels, while decreasing plasma CK and LDH concentrations. In addition, SFI treatment significantly upregulated phosphorylated AKT and eNOS, enhanced Bcl‐2 protein expression, and elevated SOD activity [144]. Notably, the cardioprotective effects of SFI were abolished by wortmannin. In summary, SFI‐mediated activation of the PI3K/AKT axis is essential for attenuating diabetic cardiac I/R injury.

#### 1.2.5. Autophagy Pathway

##### 1.2.5.1. Monomer

**FIGURE 4:**
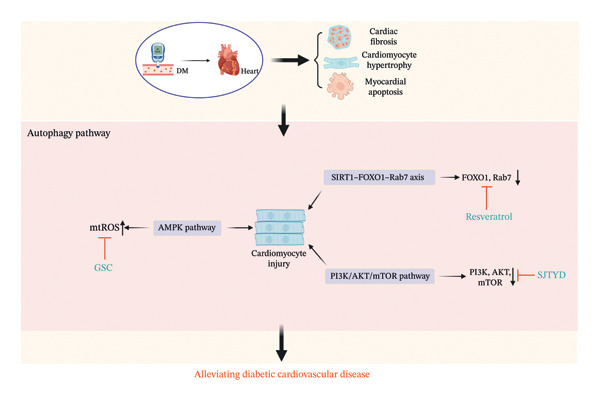
The mechanism of Chinese herbal medicine in treating diabetic cardiovascular diseases through the autophagy pathway. DM, diabetic mellitus; SIRT1, sirtuin 1; AMPK, 5′‐adenosine monophosphate–activated protein kinase; PI3K, phosphoinositide 3‐kinase; AKT, protein kinase B; FoxO1, forkhead box O1; mTOR, mammalian target of rapamycin; GSC, Ginseng‐Sanqi‐Chuanxiong; SJTYD, Shengjie Tongyu decoction.

Resveratrol has demonstrated beneficial effects in DCM, yet its role in modulating cardiac function through autophagy remains insufficiently defined. This study explored how resveratrol mitigates HF in the diabetic mice, emphasizing SIRT1 and autophagic flux [63]. STZ‐induced DCM mice treated with long‐term resveratrol exhibited ameliorated cardiac performance, reduced oxidative injury, and decreased myocardial apoptosis. Western blot analyses showed that resveratrol lowered p62 protein levels while increasing SIRT1 and Rab7 expression activity. Blocking autophagic flux increased mortality and negated resveratrol’s impact on p62, though SIRT1 and Rab7 levels remained unchanged. In H9C2 cells, excessive H_2_O_2_ elevated p62, cleaved caspase‐3, and acetylated forkhead box O1 (FOXO1), while suppressing SIRT1 [145]. Furthermore, sirtinol and SIRT1/Rab7 siRNA impaired the ability of resveratrol to promote autophagic flux [146]. Resveratrol also enhanced FOXO1 recruitment to the Rab7 promoter in a SIRT1‐dependent manner. Collectively, the findings establish the critical contribution of the SIRT1–FOXO1–Rab7 signaling axis to resveratrol‐mediated regulation of autophagic flux, offering insights into potential therapeutic strategies for DCM (Figure 4).

##### 1.2.5.2. Herbal Formula

The Ginseng‐Sanqi‐Chuanxiong (GSC), composed of *Panax ginseng* C. A. Mey., *Panax notoginseng* (Burk.) F. H. Chen, and Ligusticum chuanxiong Hort., has been used in the treatment of cardiovascular and metabolic diseases [147]. The research investigates whether GSC extracts, a form of CHM, can alleviate HAEC senescence induced by HG and palmitic acid, and explores the underlying mechanisms [64]. The results show that GSC extracts observably enhance antisenescence activity by reducing mitochondrial ROS (mtROS) levels in the senescent HAECs. In addition, they promote mitophagy, which further contributes to mtROS reduction. The extracts activate mitophagy through the AMPK pathway, and the suppression of AMPK—via pharmacological agents or genetic methods—reverses the antisenescence effects [148]. Overall, GSC extracts effectively protect against HG/palmitate‐induced endothelial senescence and mtROS accumulation through AMPK pathway‐mediated regulation of mitophagy, suggesting their therapeutic potential for vascular complications.

Shengjie Tongyu decoction (SJTYD) is a CHM commonly used to treat myocardial disorders, yet its therapeutic effects on DCM remain insufficiently defined. The research explored the impact of SJTYD on DCM, its underlying mechanisms, the role of autophagy, and the involvement of the mammalian target of rapamycin (mTOR) signaling pathway [65]. A STZ‐induced diabetic mouse model was employed to assess the cardioprotective efficacy of SJTYD. Bioinformatic analyses revealed that SJTYD observably affected lncRNA H19 and the mTOR signaling network [149]. Echocardiographic assessments using the Vevo2100 system demonstrated that SJTYD improved cardiac function in DCM mice. Histological evaluations—including Masson’s staining, TEM, and Western blotting—showed that SJTYD alleviated myocardial injury, reduced autophagosome formation, and decreased the expression of autophagy‐related proteins. Furthermore, SJTYD enhanced the phosphorylation levels of PI3K, AKT, and mTOR, while downregulating autophagy markers, including microtubule‐associated protein 1 light chain 3‐II (LC3A‐II) and Beclin‐1 [150, 151]. Western blot and immunofluorescence analyses in cardiomyocytes further revealed that the protective effects of SJTYD were potentiated by lncRNA H19 and attenuated by 3‐methyladenine. Collectively, these findings suggest that SJTYD confers protection against DCM by inhibiting cardiomyocyte autophagy via lncRNA H19‐mediated activation of the PI3K/AKT/mTOR pathway.

#### 1.2.6. Apoptosis Pathway

##### 1.2.6.1. Monomer

**FIGURE 5:**
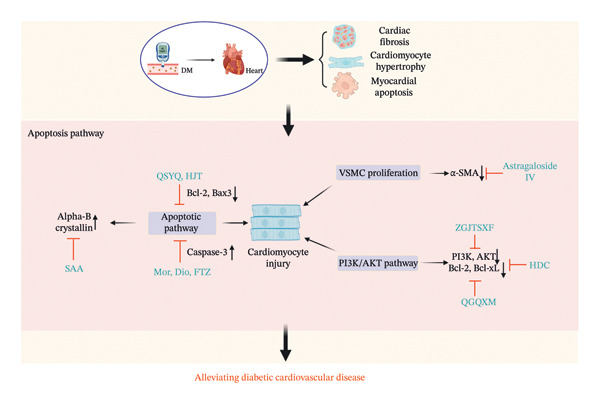
The mechanism of Chinese herbal medicine in treating diabetic cardiovascular diseases through the apoptosis pathway. DM, diabetic mellitus; Bcl‐2, B‐cell lymphoma‐2; Bax, BCL2‐associated X; Bcl‐xL, B‐cell lymphoma‐extralarge; α‐SMA, α‐smooth muscle actin; PI3K, phosphoinositide 3‐kinase; AKT, protein kinase B; VSMC, vascular smooth muscle cell; SAA, Salvianic acid A; M + D, morroniside + diosgenin; FTZ, Fufang‐Zhenzhu‐Tiaozhi capsule; HJT, Hongjingtian injection; ZGJTSXF, Zuogui Jiangtang Shuxin formula; HDC, Huangqi‐Danshen Metabolite; QGQXM, Qigui Qiangxin mixture; QSYQ, Qishen Yiqi drop pill.

Astragaloside IV, a primary bioactive compound derived from Astragalus mongholicus Bunge, is extensively employed in CHM to manage diabetes and cardiovascular disorders. Nevertheless, the impact of Astragaloside IV on vascular smooth muscle cells (VSMCs), which are of great significance in diabetic vascular complications, has not been thoroughly researched. This research investigates the effects of Astragaloside IV on VSMC proliferation, apoptosis, and phenotypic modulation under HG conditions [66]. The findings indicate that Astragaloside IV inhibits VSMC proliferation and suppresses the HG‐induced increase in the proliferation index. It also promotes apoptosis in VSMCs, as evidenced by characteristic morphological changes and a reduction in ΔΨm [152, 153]. Additionally, Western blot analysis revealed that Astragaloside IV upregulated α‐SMA expression, a marker of contractile phenotype modulation in VSMCs. Overall, Astragaloside IV appears to prevent HG‐induced VSMC proliferation by modulating the cell cycle, promoting apoptosis, and regulating VSMC phenotype, suggesting its potential to inhibit pathological vascular remodeling in diabetic patients [154] (Figure 5).

SAA is a CHM known for its beneficial effects on cardiovascular diseases, yet the mechanisms through which it improves cardiac function in DCM remain unclear. The research explored the cardioprotective effects of SAA in DCM and explored its potential mechanisms. Diabetes was induced in rats using STZ [67]. The results suggest that SAA treatment ameliorates heart function, regulates glucolipid metabolism levels, enhances mitochondrial respiration, and decreases inflammation and apoptosis [155]. Additionally, SAA inhibited the apoptotic pathway via alpha‐B crystallin in the rats with DCM.

##### 1.2.6.2. Herbal Formula

The Hedysarum multijugum Maxim.—Radix Salviae metabolite (HDC) observably ameliorates glucose and lipid metabolic dysregulation in T2DM rats with myocardial injury, while also markedly attenuating myocardial fibrosis and hypertrophy [68]. This research explored the roles of the HDC on myocardial apoptosis in rats with DCM using the network pharmacology approach [68]. Network analysis revealed that HDC influences DCM associated with biological processes, including reducing apoptosis, responding to hypoxia, regulating steroid hormone signaling, maintaining cellular iron balance, and enhancing PI3K signaling. It also affects pathways like HIF‐1, estrogen, insulin resistance, PPAR, vascular endothelial growth factor (VEGF), and PI3K‐AKT. The experiments demonstrated that HDC decreased fasting plasma glucose (FPG), HbA1c, and MDA levels while increasing SOD and GSH‐Px [156, 157]. Immunohistochemistry results indicated that HDC modulates protein expression in apoptosis pathways in the DCM rats, suggesting its potential therapeutic effect in ameliorating myocardial apoptosis.

Zuogui Jiangtang Shuxin formula (ZGJTSXF) has been shown to improve glucolipid metabolism, suppress the production of serum inflammatory factors, and protect myocardial tissue by mitigating lipid peroxidation in T2DM mice [158]. This study employed an integrated network pharmacology and experimental validation approach to elucidate the therapeutic mechanisms of ZGJTSXF in DCM [69]. Metabolomic profiling identified 78 serum metabolites in treated rats, including flavonoids, nucleosides, small peptides, phenylpropanoids, organic acids, alkaloids, iridoids, phenanthrenequinones, saponins, and phenols. The network pharmacology identified that ZGJTSXF may exert its effects through multiple targets, such as ALB, AKT1, TNF, GAPDH, VEGF‐A, EGFR, CASP3, SRC, JUN, and MAPK3, as well as through modulation of the PI3K/AKT signaling pathway [159, 160]. Experimentally, ZGJTSXF treatment significantly ameliorated DCM in mice by improving glycemic control, cardiac function, and myocardial histology while attenuating cardiomyocyte apoptosis through PI3K/AKT pathway activation and upregulation of Bcl‐2/Bcl‐xL.

Qigui Qiangxin mixture (QGQXM) has shown therapeutic benefits in DCM; however, its bioactive metabolites and mechanisms of action remain insufficiently characterized. This study sought to clarify these aspects using a combination of network pharmacology and experimental verification [70]. UPLC‐Q/TOF‐MS characterization of QGQXM revealed bioactive metabolites, which were subsequently subjected to network pharmacology analysis to elucidate their potential therapeutic mechanisms against DCM. In vivo evaluation was conducted in a DCM model induced by STZ and HFD. A total of 25 circulating metabolites were identified, corresponding to 121 DCM‐associated targets. Key predicted targets included GAPDH, AKT1, TNF, PPARG, CASP3, EGFR, and HIF1 [161]. Pathway enrichment analysis suggested that QGQXM may exert cardioprotective effects by modulating signaling pathways, including PI3K/AKT, mTOR, MAPK, insulin signaling, insulin resistance, and apoptosis [162]. Experimental results confirmed that QGQXM improved cardiac function, reduced myocardial fibrosis, and suppressed myocardial apoptosis in DCM rats. Furthermore, QGQXM exerted antiapoptotic effects by activating the PI3K/AKT pathway, upregulating Bcl‐2, and suppressing caspase‐9 activity.

The FTZ capsule has been reported to exert cardioprotective effects in various cardiovascular conditions, yet its specific mechanisms remain to be fully clarified. This study examined the distinct protective actions of FTZ in minipigs with DM‐CHD and coronary AS [71]. Cardiac function, coronary artery stenosis, and plaque were evaluated using ultrasonography, electrocardiography, and imaging techniques. Porcine tissues and HUVECs—the latter treated with HG or FTZ—were subsequently analyzed. FTZ markedly improved glucose and lipid metabolism, showing therapeutic efficacy comparable to metformin combined with Atorvastatin (M + A). Both FTZ and M + A improved myocardial injury. In the coronary arteries of DM‐CHD minipigs, phosphorylation of IκB and NF‐κB was elevated, accompanied by increased expression of IL‐1β, Bax, cleaved caspase‐3, Bcl‐2, and α‐SMA, along with reduced levels of CD31 and VE‐cadherin [163]. FTZ treatment reversed these molecular alterations similarly to M + A. Additionally, FTZ reduced HG‐induced endothelial cell injury and aberrant migration in HUVECs. Collectively, these findings indicate that FTZ mitigates coronary atherosclerosis progression by suppressing apoptosis [121, 164], thereby offering protective benefits in DM‐CHD.

*Cornus officinalis* Siebold and Zucc. and *Dioscorea oppositifolia* L. are commonly used CHMs for managing DM and its complications. Morroniside (Mor), derived from *Cornus officinalis,* and diosgenin (Dio), extracted from *Dioscorea oppositifolia*, were combined to form a novel formula termed M + D. The research explored the impact of M + D on DCM, with a particular focus on its ability to suppress caspase‐3 protein expression, and compared its efficacy with that of Mor, Dio, and metformin [72]. The research evaluated cell viability, apoptosis, and the expression levels of Bax, Bcl‐2, and caspase‐3 in the rat cardiomyocytes. The findings showed that Mor, Dio, and M + D enhanced cell viability and reduced apoptosis. They also regulated Bax and Bcl‐2 expression and significantly reduced caspase‐3 levels, with M + D demonstrating the strongest effects [165]. Overall, Mor, Dio, and M + D effectively mitigated hyperglycemia‐induced myocardial damage by decreasing apoptosis in rat cardiomyocytes.

#### 1.2.7. Chinese Patent Medicine

Qishen Yiqi drop pill (QSYQ) has demonstrated cardioprotective properties in various cardiovascular conditions; however, its effects on diabetes‐related myocardial injury remain insufficiently understood. This research investigated the ability of QSYQ to mitigate HG‐induced damage in H9c2 cardiomyocytes [73]. The results showed that QSYQ markedly improved cell viability and reduced cytotoxicity. Quantification of apoptotic markers (caspase‐3, Bcl‐2, Bax, p53) demonstrated QSYQ’s potent attenuation of HG‐induced apoptosis in HUVECs. Moreover, QSYQ effectively decreased ROS production. Further analysis revealed that QSYQ maintained ΔΨm and suppressed mPTP opening, directly preserving mitochondrial function in HG‐stressed cells [166, 167].

Despite the widespread clinical application of Hongjingtian injection (HJT) in managing vascular pathologies—particularly diabetic angiopathies (DA)—the precise molecular mechanisms governing its therapeutic effects remain elusive. The research intended to explore the underlying mechanisms of HJT via network pharmacology [74]. In this study, A7r5 VSMCs exposed to HG were utilized as an in vitro model to investigate apoptosis mechanisms. The participation of key molecular targets and the AKT signaling pathway was confirmed through Western blot analysis, utilizing both pharmacological inhibition (LY294002) and activation (SC79) of AKT. The network analysis identified 10 targets and 15 pathways associated with apoptosis in DA. Experimental findings demonstrated that HJT significantly increased apoptosis in hyperglycemia‐induced VSMCs, consistent with the network predictions. HJT decreased the levels of *p*‐AKT, MMP9, and PCNA, while upregulating p53 and cleaved caspase‐3 and elevating the Bax/Bcl‐2 ratio [168]. Furthermore, SC79‐mediated AKT activation partially attenuated HJT‐induced effects in VSMCs, further supporting AKT pathway involvement [169]. Taken together, these findings systematically delineate the mechanistic basis of HJT’s action in DA, demonstrating its capacity to induce apoptosis in HG‐stimulated VSMCs via targeted inhibition of the AKT signaling cascade.

#### 1.2.8. Others’ Pathways

##### 1.2.8.1. Monomer

**FIGURE 6:**
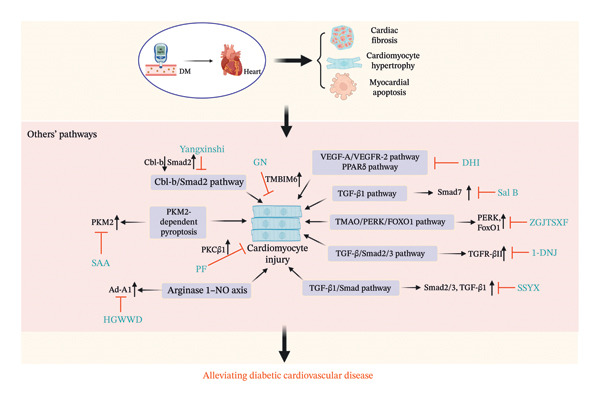
The mechanism of Chinese herbal medicine in treating diabetic cardiovascular diseases through others’ pathways. DM, diabetic mellitus; Cbl‐b, casitas B lymphoma‐b; smad2, mothers against decapentaplegic homolog 2; TMBIM6, transmembrane BAX inhibitor motif containing 6; PKCβ1, protein kinase Cβ1; Ad‐A1, adenoviral arginase 1; TMAO, trimethylamine‐N‐oxide; FoxO1, forkhead box O1; PERK, protein kinase RNA–like endoplasmic reticulum kinase; TGF‐β1, transforming growth factor‐β1; VEGF‐A, vascular endothelial growth factor A; VEGFR‐2, VEGF receptor‐2; PPARδ, peroxisome proliferator–activated receptor δ; Smad7, recombinant mothers against decapentaplegic homolog 7; TGFR‐βII, TGF‐β receptor II; PKM2, pyruvate kinase M2; PF, Paeoniflorin; Sal B, Salvianolic acid B; SAA, salvianic acid A; 1‐DNJ, 1‐deoxynojirimycin; HGWWD, Huangqi Guizhi Wuwu decoction; GN, Ginseng Dingzhi decoction; SSYX, Shensong Yangxin capsule; DHI, Danhong injection; ZGJTSXF, Zuogui Jiangtang Shuxin formula.

Salvianolic acid B (Sal B), an active metabolite derived from *Salvia miltiorrhiza* Bunge, is widely applied in CHM for cardiovascular treatments. Accumulating evidence indicates that Sal B attenuates DCM progression by suppressing key fibrotic pathways in cardiac tissue. The research explored the protective effects of Sal B on DCM‐induced myocardial fibrosis and clarified its underlying mechanisms [75]. Animal tests suggested that Sal B observably ameliorated cardiac function, suppressed collagen deposition and phenotypic transformation, and alleviated myocardial fibrosis by upregulating Recombinant Mothers Against Decapentaplegic Homolog 7 (Smad7), thereby suppressing the TGF‐β1 signaling pathway. Additionally, in vitro assays demonstrated that Sal B effectively reduced the proliferation, migration, phenotypic switching, and collagen production of cardiac fibroblasts under HG conditions. Sal B also decreased Smad7 ubiquitination, stabilizing its protein expression and further inhibiting TGF‐β1 pathway activation [170, 171]. This mechanism likely underpins the ability of Sal B to mitigate myocardial fibrosis in DCM. In conclusion, Sal B ameliorates DCM‐induced myocardial fibrosis by promoting Smad7 deubiquitination, stabilizing its expression, and suppressing the TGF‐β1 signaling pathway (Figure 6).

Bombyx Batryticatus (BB) has been used for centuries in traditional medicine for its antispasmodic effects and its ability to promote blood circulation. Recent studies have focused on myocardial fibrosis and cardiomyocyte hypertrophy associated with aberrant N‐glycosylation of key myocardial proteins [76]. To investigate early‐stage N‐glycosylation alterations in diabetic myocardium and evaluate the therapeutic efficacy of 1‐deoxynojirimycin (1‐DNJ), a bioactive compound isolated from BB, a db/db diabetic mouse model was employed. Hydrophilic interaction chromatography, solid‐phase extraction, and LC–MS were applied to characterize site‐specific N‐glycosylation patterns in left ventricular cardiomyocyte proteins. Results revealed that 1‐DNJ significantly reduced N‐glycosylation of myocardium proteins in the db/db mice [172]. Furthermore, 1‐DNJ treatment decreased serum markers and fibrosis‐related factors. Notably, an elevation in α‐1,6‐fucosylation of myocardial glycans was observed in db/db mice, and 1‐DNJ substantially modulated this modification [76]. The study also explored the TGF‐β/Smad2/3 signaling pathway and conducted semiquantitative analysis of core α‐1,6‐fucosylated TGF‐β receptor II (TGFR‐βII) via Western blotting [173], with findings corroborated by lens culinaris agglutinin (LCA) histochemistry and lectin blot analysis. While 1‐DNJ did not significantly alter α‐1,6‐fucosyltransferase (FUT8) mRNA expression, it effectively reduced N‐acetylglucosamine (N‐GlcNAc) levels and substrate concentration, alleviating fibrosis associated with DCM.

*Salvia miltiorrhiza* Bunge contains SAA, a key water‐soluble phenolic acid, widely used in CHM and health supplements. SAA has been shown to alleviate diabetic complications, in part by delaying early AS progression through modulation of the NLRP3 inflammasome. This study further explored the protective role of SAA in ECs by focusing on its regulation of pyruvate kinase M2 (PKM2) [77]. In STZ‐induced diabetic ApoE^−/−^ mice, SAA treatment markedly reduced atherosclerotic plaque burden and improved vascular pathology. SAA also effectively suppressed NLRP3 inflammasome activation and attenuated pyroptotic cell death [174, 175]. Mechanistic analyses identified PKM2 as a pivotal molecular target of SAA. Specifically, SAA was found to bind to the allosteric activator pocket of PKM2, inhibit Y105 phosphorylation, and block its nuclear translocation. In addition, SAA reduced HG‐induced lactate overproduction and partially diminished lactate‐mediated PKR phosphorylation, a key regulatory step in NLRP3 inflammasome signaling [175, 176]. Further experiments using phenylalanine supported that the antipyroptotic effects of SAA under HG conditions depend on PKM2 modulation [77, 177]. Complementary assays employing c16, a PKR inhibitor, together with phenylalanine, indicated that the regulation of PKR phosphorylation contributes to SAA’s ability to modulate PKM2‐dependent pyroptosis.

Paeoniflorin (PF), recognized for its antiapoptotic, anti‐inflammatory, and antithrombotic properties, exerts significant protective effects in cardiovascular and cerebrovascular disorders. This study explored PF’s potential protective effects on HUVECs exposed to fluctuating glucose levels in vitro and on diabetic rats in vivo [ [78], p. 1]. In HUVECs, intermittent glucose changes over 8 days led to increased apoptosis, inflammation, oxidative stress, and elevated PKCβ1 protein levels, which PF successfully mitigated. LY333531, a PKCβ1 inhibitor, and metformin showed similar outcomes. In diabetic rats with fluctuating hyperglycemia, PF also protected against vascular damage, mirroring in vitro results [178]. Overall, PF reduces vascular injury from variable hyperglycemia by inhibiting oxidative stress, reducing inflammation, and suppressing PKCβ1 protein levels.

##### 1.2.8.2. Herbal Formula

In clinical practice, ZGJTSXF has been employed for the treatment of DCM for many years. However, the mechanisms by which ZGJTSXF modulates gut microbiota and metabolic processes to prevent or alleviate DCM remain insufficiently understood. This study utilized DCM mouse models to explore the roles of ZGJTSXF [79]. The results indicated that ZGJTSXF notably improved DCM‐related symptoms through regulating the gut‐heart axis. Specifically, ZGJTSXF treatment improved glycolipid metabolism, enhanced cardiac function and morphology, and reduced myocardial apoptosis in DCM mice. Additionally, Z ZGJTSXF exerted its therapeutic effects by modulating the trimethylamine‐N‐oxide (TMAO)/protein kinase RNA‐like endoplasmic reticulum kinase (PERK)/FOXO1 signaling pathway [179, 180]. In summary, ZGJTSXF ameliorated DCM in mice by reducing TMAO levelsand inhibiting activation of the TMAO/PERK/FOXO1 pathway.

Yangxinshi, a Chinese medicine formula, is utilized for treating cardiovascular diseases. To better elucidate its potential antifibrotic effects in diabetes, it is important to identify its active metabolites, therapeutic targets, and related molecular pathways through pharmacological network analysis. This study employed an integrated approach combining network pharmacology prediction with experimental validation to elucidate the therapeutic effects of Yangxinshi against HG‐induced CFs [80]. Key proteins, including casitas B‐lineage lymphoma‐b (Cbl‐b), α‐SMA, and phosphorylated Mothers Against Decapentaplegic Homolog 2 (p‐Smad2), were assessed. These findings demonstrated that Yangxinshi significantly regulated CF activation in DCM, likely via modulation of the Cbl‐b–mediated signaling pathway. Specifically, Yangxinshi treatment was related to an increase in Cbl‐b expression and a reduction in p‐Smad2 and α‐SMA levels, which supported the predictions made via network pharmacology [181]. In conclusion, the protective effects of Yangxinshi on cardiac fibrosis appear to be associated with modulation of the Cbl‐b/Smad2 signaling pathway, providing new insights into its therapeutic potential for managing fibrosis in DCM.

Ginseng Dingzhi decoction (GN), a traditional Chinese herbal remedy, has demonstrated cardioprotective effects in HF, possibly through modulation of the gut microbiota and maintenance of mitochondrial homeostasis. Nevertheless, the specific mechanisms linking GN to microbial regulation and mitochondrial function are not yet fully defined. GN has been found to adjust microbial populations, increasing short‐chain fatty acids and beneficial bacteria while decreasing harmful pathogens associated with diabetes [81]. This intervention notably reduces heart failure and myocardial hypertrophy, enhances heart function, and preserves mitochondrial balance and redox status in myocardial cells under HG conditions. In vitro assays further showed that the protective actions of GN on cellular injury and mitochondrial homeostasis were abolished upon TMBIM6 knockdown via siRNA, indicating that TMBIM6 plays a crucial role in GN‐mediated regulation of mitophagy and mitochondrial stability [81, 182, 183]. Additionally, combining GN with metformin in HG‐treated cardiomyocytes improved mitophagy levels and offered cardioprotection.

Huangqi Guizhi Wuwu decoction (HGWWD) is a traditional medicine formula used to treat cardio‐cerebrovascular diseases associated with vascular pathology. The research explored the roles of HGWWD on vascular dysfunction induced by STZ in the mouse [82, p. 1]. In vivo experiments included wild‐type mice and EC‐specific arginase 1 knockout mice (EC‐A1^−/−^), divided into control, diabetic, and HGWWD‐treated diabetic groups over 2 weeks. In vitro, aortic tissues were exposed to HGWWD‐enriched mouse serum, with or without adenoviral arginase 1 (Ad‐A1) transduction. HGWWD treatment increased NO levels in the aorta and plasma, and also reduced aortic arginase 1 and endothelial arginase activity expression levels [184]. Notably, the effects of HGWWD were reversed by an NO synthesis inhibitor. Furthermore, the protection of HGWWD serum against endothelial dysfunction was obstructed by Ad‐A1 transduction. Overall, the findings indicate that HGWWD ameliorates vascular dysfunction primarily through modulation of the arginase 1–NO signaling axis, with endothelial arginase 1 serving as a key therapeutic target.

##### 1.2.8.3. Chinese Patent Medicine

Shensong Yangxin capsule (SSYX) is an established antiarrhythmic agent in Chinese clinical practice, yet its potential modulatory effects on interstitial fibrosis in DCM remain incompletely characterized. This research investigated the effect of SSYX on myocardial fibrosis in diabetic rats [83]. Specifically, STZ‐induced diabetic rats on an HFD were examined to evaluate SSYX’s antifibrotic effects. The experimental data demonstrated that SSYX treatment significantly decreased the HW/BW and improved LVEF in T2DM rats. Histological analyses—including HE staining, TEM, and Masson staining—demonstrated that SSYX attenuated collagen deposition and myocardial fibrosis. Moreover, SSYX treatment decreased the mRNA expression levels of collagen III (col‐3), collagen I (col‐1), TGF‐β1, MMP‐2, and MMP‐9, while upregulating Smad7 expression [185]. Protein analysis revealed reduced levels of p‐Smad2/3 and TGF‐β1, alongside elevated Smad7 expression. In conclusion, SSYX appears to alleviate cardiac fibrosis and ameliorated cardiac function via suppressing activation of the TGF‐β1/Smad signaling pathway.

Danhong injection (DHI) is a commonly used for cardiovascular issues, yet its mechanism of action is still not clear. The research explored DHI’s therapeutic potential in induced and genetic T2DM mice with peripheral arterial disease [84]. Diabetic mice demonstrated significantly impaired perfusion recovery in the hind‐limb ischemia (HLI) model. DHI treatment markedly restored limb perfusion, concurrently reducing HG and enhancing insulin sensitivity. Additionally, DHI treatment elevated levels of VEGF‐A and its receptor, VEGFR‐2, in the ischemic muscle. The IPA confirmed interactions between the peroxisome proliferator–activated receptor δ (PPARδ)/peroxisome proliferator–activated receptor γ (PPARγ) and VEGF‐A/VEGFR‐2 pathways [186, 187]. The study demonstrated that DHI‐induced VEGF‐A/VEGFR‐2 upregulation enhanced PPARδ expression in T2DM. Overall, these findings indicate that DHI promotes blood flow recovery by stimulating angiogenesis and improving glucose metabolism through activation of both VEGF‐A/VEGFR‐2 signaling and PPARδ pathways.

### 1.3. Therapeutic Mechanism of CHM in Treating Diabetic Cerebrovascular Diseases

#### 1.3.1. Intracellular Inflammatory Pathway

##### 1.3.1.1. Monomer

**FIGURE 7:**
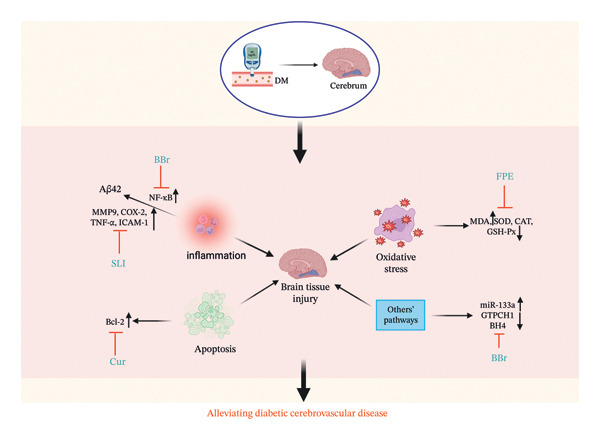
Therapeutic mechanism of Chinese herbal medicine in treating diabetic cerebrovascular diseases. DM, diabetic mellitus; NF‐κB, nuclear factor‐kappa B; COX‐2, cyclooxygenase 2; ICAM‐1, intercellular adhesion molecule‐1; TNF‐α. Tumor necrosis factor alpha; MMP9, matrix metalloproteinase 9; MDA, malondialdehyde; SOD, superoxide dismutase; CAT, catalase; GSH‐Px, glutathione peroxidase; Bcl‐2, B‐cell lymphoma‐2; Cur, curcumin; BBr, berberine; SLI, salvianolate lyophilized injection; FPE, *Flos Puerariae* extract.

Berberine (BBr) exhibits well‐documented multifunctional pharmacological properties, including anti‐inflammatory, antimicrobial, hypoglycemic, hypolipidemic, antioxidant, antitumorigenic, and neuroprotective activities [188]. In the diabetic brain, insulin resistance and chronic inflammation lead to the production of the β‐amyloid 42 (Aβ42), a hallmark of Alzheimer’s disease. To explore the impact of BBr on cognitive impairment in DM, Chen et al. investigated its potential to ameliorate insulin resistance and alleviate inflammation in the diabetic rats [85]. BBr was administered intragastrically, and cognitive function was evaluated using a fear‐conditioning test. Protein expression was assessed by Western blotting, while glucose uptake in the prefrontal cortex was quantified via positron emission tomography (PET). Inflammation mediators were measured using ELISA kits. The study demonstrated that BBr inhibited insulin resistance and inflammation in the medial prefrontal cortex (mPFC) of diabetic rats. It also downregulated the MAPK and PI3K/AKT/mTOR signaling pathways, along with the isoforms PKCη and PKCε, and inhibited NF‐κB translocation in neurons [189, 190]. Moreover, the neuron‐specific glucose transporter GLUT3 was observably upregulated, enhancing cerebral glucose uptake. BBr further reduced the expression of beta‐site amyloid precursor protein‐cleaving enzyme 1 (BACE‐1) and amyloid precursor protein, thereby decreasing oligomeric Aβ42 production. Collectively, these mechanisms improved cognitive function and information processing (Figure 7).

##### 1.3.1.2. Chinese Patent Medicine

Salvianolate lyophilized injection (SLI) exhibits robust antioxidant capacity and strong free radical–scavenging activity, and its pharmacological benefits include enhanced cerebral blood flow, reduction of infarct volume, and modulation of the renin–angiotensin system [191, 192]. Clinical and experimental evidence indicates that DM increases the risk of recurrent ischemic stroke and contributes to higher post‐stroke mortality. Previous studies have also shown that SLI reduces infarct size in diabetic rat models [193]. This research aims to elucidate the mechanisms through which SLI influences stroke outcomes in rats with Type 1 DM (T1DM) [86]. A T1DM model was established in male Wistar rats via STZ administration, followed by the induction of middle cerebral artery occlusion (MCAO). Micro‐CT imaging was used to characterize cerebrovascular alterations in the ischemic region, while PET/CT was employed to evaluate cerebral glucose uptake. Fourteen days after MCAO, immunofluorescence staining and Western blot analyses were performed to quantify the expression of RAGE, matrix metalloproteinase 9 (MMP9), inflammatory mediators (COX‐2, TNF‐α, ICAM‐1), as well as HQ‐1, HO‐1, and Nrf2 [194, 195]. SLI markedly promoted microvascular formation within ischemic brain areas and significantly increased glucose uptake [196]. Additionally, SLI significantly downregulated the expression of RAGE, MMP9, and proinflammatory markers, while upregulating HO‐1, HQ‐1, and Nrf2. Collectively, these findings suggest that SLI confers neuroprotection in T1DM rats subjected to MCAO, likely through the suppression of inflammatory responses and activation of the Nrf2/HO‐1 signaling pathway.

#### 1.3.2. Oxidative Stress Pathway

##### 1.3.2.1. Single Medicinal Botanical Drug

The impact of *Flos Puerariae* extract (FPE) on diabetes‐associated cognitive dysfunction was evaluated using the Morris water maze (MWM), a well‐established behavioral test for assessing spatial learning and memory capabilities [87]. Additional measurements included body weight, blood glucose levels, FFA, total cholesterol (TCH), oxidative stress indicators (MDA, SOD, CAT, and GSH‐Px), along with acetylcholinesterase (AChE) activity in both the cerebral cortex and hippocampus [197]. Oral administration of FPE produced significant therapeutic effects in STZ‐induced diabetic mice [198], attenuating cognitive deficits while simultaneously stabilizing body weight and ameliorating both dyslipidemia and hyperglycemia. Moreover, FPE treatment significantly reduced oxidative stress markers (MDA levels), enhanced antioxidant enzyme activities (CAT, GSH‐Px), and inhibited AChE activity. Collectively, these findings suggest that FPE ameliorates diabetes‐related cognitive dysfunction primarily by mitigating oxidative stress and regulating AChE activity.

#### 1.3.3. Apoptosis Pathway

##### 1.3.3.1. Monomer

*Curcuma longa* L. (Cur) is widely recognized for its anti‐inflammatory, antioxidant, antiviral, antifibrotic, anticoagulant, and glucose‐regulatory properties. In this study, Cur administration markedly improved neurological function scores, reduced cerebral infarct volume, and mitigated cerebral edema following MCAO [88]. Cerebral GLUT1 and GLUT3 expression was markedly reduced following MCAO but showed significant restoration after Cur treatment [199]. Cur further attenuated neuronal apoptosis, highlighting its neuroprotective and antiapoptotic capabilities. To determine whether Cur exerts its antiapoptotic effects through the regulation of GLUT1 and GLUT3, siRNAs were used to silence these transporters. Knockdown of GLUT1 and GLUT3 led to reduced expression of both transporters as well as decreased Bcl‐2 levels, even in the presence of Cur. The results indicate that Cur confers neuroprotection against apoptosis and ischemic injury predominantly through the upregulation of GLUT1 and GLUT3.

#### 1.3.4. Others’ Pathways

##### 1.3.4.1. Monomer

BBr has been extensively documented to exhibit broad‐spectrum pharmacological activities, including significant antimicrobial effects against fungal and bacterial/viral pathogens, potent antitumor properties, as well as promising therapeutic potential in diabetes management [200]. Dysregulation of miR‐133a in ECs is associated with diabetes‐related endothelial dysfunction [201]. However, the effectiveness of BBr, a natural metabolite isolated from Coptis chinensis, in treating diabetes‐induced vascular dementia remains unclear. In this study, rats received five consecutive STZ injections to induce diabetes and vascular dementia [89]. miR‐133a expression levels were precisely quantified using fluorescence in situ hybridization, while cognitive functions—particularly learning and memory—were comprehensively evaluated through a combination of behavioral paradigms, including step‐down avoidance, step‐through avoidance, and MWM tests. The findings demonstrated that STZ‐induced HG significantly upregulated miR‐133a expression in vascular ECs while downregulating GTPCH1 mRNA levels and reducing BH4 content, all of which were effectively reversed by BBr treatment. HG significantly impaired endothelium‐dependent vasorelaxation and reduced cerebral blood flow, both of which were effectively reversed by BBr treatment. In vitro, miR‐133a agomirs abolished BBr‐induced vasodilation, while L‐sepiapterin prevented endothelial dysfunction in middle cerebral arteries. Furthermore, BBr administration significantly ameliorated STZ‐induced cognitive impairment in rats while downregulating miR‐133a expression and elevating both BH4 levels and NO production in HG‐exposed ECs [202].

### 1.4. Limitations

The included studies have certain limitations that should be acknowledged. (1) The pharmacological efficacy and chemical composition of CHMs may vary due to factors such as growing environment, harvest season, and postharvest processing techniques. Notably, geoauthentic herbs often exhibit superior bioactive compound profiles compared to nonauthentic sources. (2) The manufacturing process of CHM preparations involves multiple critical steps, including extraction, purification, concentration, and formulation, each of which can influence the stability of the final product through specific process parameters. (3) Animal models cannot fully replicate the complex pathophysiology of human diseases. Interspecies physiological differences may lead to discrepancies in the efficacy and mechanisms of CHM between animal studies and clinical applications. Future studies should be conducted in accordance with guideline standards [203].

## 2. Conclusion

This review provides a brief overview of the mechanisms underlying diabetic cardio‐cerebrovascular disease, with a particular focus on metabolism, inflammation, and oxidative stress. We then thoroughly described the CHMs used for diabetic cardio‐cerebrovascular disease, including herbal formula, monomer, single medicinal botanical drug, and Chinese patent medicine. They have demonstrated roles like antifibrosis, anti‐inflammatory, antioxidation, antiapoptotic, proangiogenesis, and metabolic adjustment. With years of clinical use, Chinese medicine has proven highly effective in treating diabetes‐induced cardiovascular and cerebrovascular diseases. Meanwhile, CHM has demonstrated multiple advantages, including multilevel, multitarget, and personalized approaches, in the treatment of diabetic cardio‐cerebrovascular disease. However, these studies often oversimplify the complex pathological processes of diabetic cardiovascular and cerebrovascular diseases, which involve multiorgan and multisystem interactions. The limitations of animal experiments are particularly pronounced, as commonly used mouse models fail to replicate the chronic progression characteristics of human diseases and inadequately reflect the metabolic heterogeneity observed in clinical practice. Clinical research also faces significant challenges, with large‐scale trials like the ACCORD study revealing an unexpected nonlinear relationship between intensive glycemic control and cardiovascular benefits, suggesting that the “metabolic memory” effect and hypoglycemia risks may have been substantially underestimated. Future investigations should employ systems biology approaches to integrate novel technologies such as single‐cell sequencing and spatial transcriptomics for in‐depth analysis of interorgan communication mechanisms. Concurrently, researchers must fully leverage real‐world data to address the temporal and spatial limitations of clinical trials, while actively exploring the therapeutic potential of the emerging interdisciplinary field of cardiometabolic medicine. Only by adopting a holistic perspective of complex systems and combining multiomics technological innovations with clinical big data mining can we overcome the current fragmentation in research and ultimately achieve precise and personalized prevention and treatment strategies for diabetic cardiovascular and cerebrovascular complications.

## Author Contributions

Xinyu Yang and Zhen Xing defined the research theme. Cuicui Cheng, Wei Li, and Shuwen Luo searched for related articles. Cuicui Cheng, Wei Li, and Shuwen Luo collated all related articles. Xinyu Yang, Ying Su, and Zhen Xing wrote the manuscript. All authors commented on the manuscript.

## Funding

The work was supported by the National Natural Science Foundation of China (Grant No. 82205088).

## Ethics Statement

The authors have nothing to report.

## Consent

The authors have nothing to report.

## Conflicts of Interest

The authors declare no conflicts of interest.

## Data Availability

Data sharing is not applicable to this article as no datasets were generated or analyzed during the current study.
